# Cerebrospinal fluid extracellular vesicle-derived miR-9-3p in spinal cord injury with neuroprotective implications and biomarker development

**DOI:** 10.1038/s42003-025-08947-3

**Published:** 2025-10-27

**Authors:** Tomoharu Tanaka, Satoru Morimoto, Keitaro Ito, Kaori Yasutake, Chris Kato, Munehisa Shinozaki, Kota Suda, Takeshi Maeda, Yoshiyuki Yato, Masaya Nakamura, Hideyuki Okano, Narihito Nagoshi

**Affiliations:** 1 Department of Orthopaedic Surgery, Keio University School of Medicine, Tokyo, Japan; 2 Keio University Regenerative Medicine Research Center, Kanagawa, Japan; 3 Department of Orthopaedic Surgery, Hokkaido Spinal Cord Injury Center, Hokkaido, Japan; 4 Department of Orthopaedic Surgery, Spinal Injuries Center, Fukuoka, Japan; 5 Department of Orthopaedic Surgery, National Hospital Organization, Murayama Medical Center, Musashimurayama, Tokyo, Japan

**Keywords:** miRNAs, Molecular neuroscience

## Abstract

Spinal cord injury (SCI) often results in severe disability, and early detection of molecular changes is crucial for guiding treatment. In both rat and human samples, we observed a significant increase in cerebrospinal fluid (CSF)-derived extracellular vesicle (EV) miR-9-3p after SCI, prompting further investigation into its role. In a rat model, miR-9-3p levels were significantly lower at the injured spinal levels but higher in the motor cortex, where astrocytes showed the highest expression. Functional analyses revealed that miR-9-3p regulates energy metabolism, immune activity, and oxidative stress in neurons, inducing transcriptional changes suggestive of stress adaptation and synaptic remodeling. These findings demonstrate that EV-associated miR-9-3p modulates injury responses by reducing energy demands and supporting structural and functional adaptation, establishing it as a promising biomarker and therapeutic target for acute SCI.

## Abstract (web summary)

Profiling of extracellular vesicle-derived miR-9-3p in cerebrospinal fluid after spinal cord injury reveals its potential role as a biomarker and regulator of acute neuroprotective responses.

## Introduction

Spinal cord injury (SCI) is a debilitating condition that causes severe motor and sensory dysfunction, leading to a marked decline in patients’ quality of life and functional independence^1^. Although various therapeutic strategies, including stem cell transplantation, anti-inflammatory drugs, and neuroprotective agents, have been developed for the acute phase of SCI^2–4^, none have demonstrated sufficient efficacy^5^. One primary reason for this shortfall is the incomplete understanding of SCI pathophysiology.

A more comprehensive understanding of SCI pathology requires detailed insights into genetic alterations. Notably, approximately 8800 genes exhibit altered expression after SCI^6^. One factor contributing to these shifts is post-transcriptional regulation, which is primarily mediated by microRNAs (miRNAs)^7,8^. miRNAs regulate cellular states and functions by inhibiting the translation of multiple target mRNAs, potentially affecting 20–30% of human genes^9^. Through these mechanisms, miRNAs are crucial for maintaining cellular homeostasis and function^10^, and their dysregulation has been implicated in many diseases^11–13^.

Simultaneously, extracellular vesicles (EVs), including exosomes and microvesicles, have emerged as important modulators of various pathological conditions^14–16^. EVs are small particles (approximately 30–150 nm in diameter)^17^ that are secreted by nearly all cell types. They encapsulate biological cargo, such as miRNAs, mRNAs, and proteins, reflecting the functional state of their parent cells^18^. By mediating intercellular communication^19^ and altering their cargo in response to disease conditions^20^, EVs provide a unique window into disease pathogenesis and offer opportunities for biomarker discovery^21^. Among the various EV cargoes, miRNAs play significant roles in conditions ranging from cancer to neurodegenerative and inflammatory disorders^22–24^, making them prime targets for investigating disease mechanisms.

Despite the potential of EV-associated miRNAs, research on EV-associated miRNAs in SCI remains limited. Most studies have focused on miRNAs within blood-derived EVs^25–27^, which primarily capture systemic rather than injury-specific inflammatory responses. Consequently, the exact cellular origins and functional roles of plasma EV miRNAs are not fully understood^28^. In contrast, cerebrospinal fluid (CSF)-derived EV miRNAs, which are situated closer to the lesion, are considered to reflect SCI pathology more accurately, thereby offering greater potential as clinically relevant biomarkers. However, few studies have examined CSF-derived EVs in patients with SCI. One report documented elevated levels of inflammasome proteins in CSF EVs from patients with SCI^29^, whereas another showed that CSF EVs activated the PI3K/AKT pathway to promote vascular regeneration in a porcine SCI model^30^. However, comprehensive miRNA profiling or detailed functional analyses of CSF-derived EVs was not performed in these studies, leaving critical gaps in our understanding of the roles of CSF-derived EVs in SCI. Moreover, to date, no study has identified a CSF-derived EV miRNA that predicts spontaneous neurological recovery after SCI in clinical samples.

To address this critical gap, we established a reliable method for collecting CSF from a rat model of SCI. We then performed a comprehensive miRNA analysis of EVs derived from both the CSF and blood, identifying several candidate miRNAs of interest. Using CSF samples from patients with SCI, we evaluated the translational relevance of these findings and explored their potential clinical applications. Moreover, we investigated the functional significance of these miRNAs to elucidate their pathophysiological impact on SCI.

## Results

### Isolation of CSF- and plasma-derived EVs

**Fig. 1:**
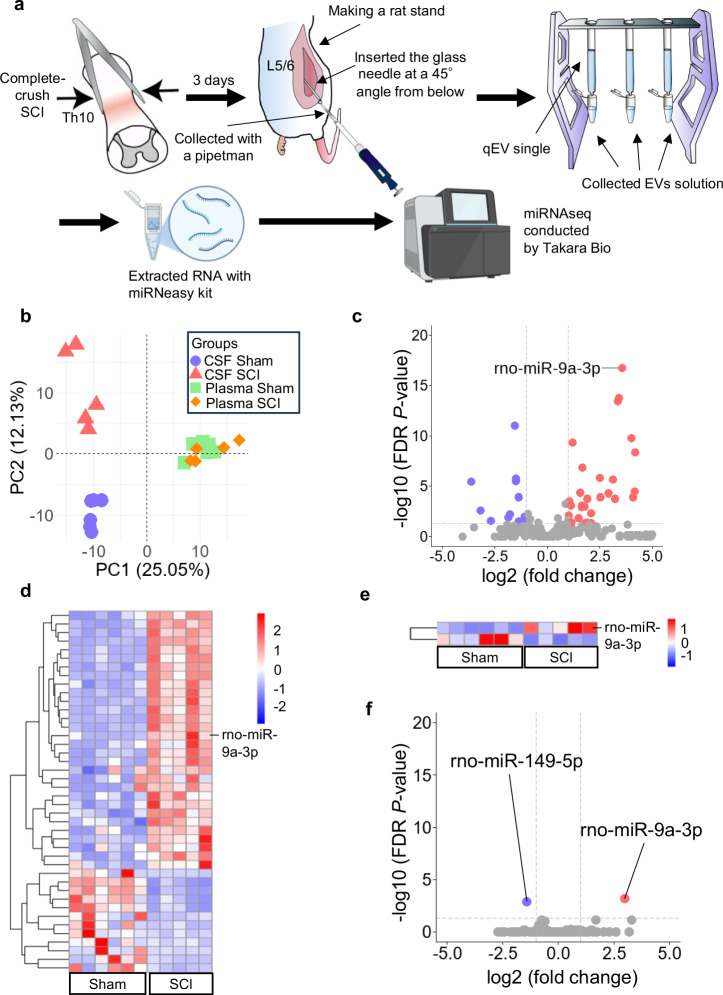
Overview of the experimental design and differential miRNA expression analysis in CSF- and plasma-derived EVs. **a** Schematic representation of the experimental workflow illustrates the rat SCI model, CSF collection, EV isolation, RNA extraction, and miRNA sequencing. The final two illustrations were created with BioRender.com. Tanaka, T. (2025) https://BioRender.com/dju0zki; https://BioRender.com/hk6zb5y. **b** The PCA score plot of CSF- and plasma-derived EV miRNAs in sham and SCI rats displays PC1 and PC2, showing distinct clustering between the sham and SCI groups, particularly in CSF. Each dot represents an independent sample (CSF sham, red triangles; CSF SCI, blue circles; plasma sham, green squares; plasma SCI, orange diamonds; sham, n = 6; SCI, n = 5). **c** Volcano plot of differentially expressed miRNAs in CSF-derived EVs, where the x-axis indicates the log2 fold change, and the y-axis represents the −log10-transformed FDR *P* value. Red dots represent significantly upregulated miRNAs, whereas blue dots represent significantly downregulated miRNAs (fold change ≥2, FDR *P* < 0.05). miR-9a-3p, the most significantly upregulated miRNA, is labeled. **d** Heatmap of 42 differentially expressed miRNAs in CSF-derived EVs displays the log2-transformed expression values, with red indicating high expression and blue indicating low expression (fold change ≥2, FDR *P* < 0.05). **e** Volcano plot of differentially expressed miRNAs in plasma-derived EVs, where the x-axis indicates log2 fold change, and the y-axis represents −log10-transformed FDR *P* value. Red dots represent significantly upregulated miRNAs, whereas blue dots represent significantly downregulated miRNAs (fold change ≥2, FDR *P* < 0.05). miR-9a-3p is among the significantly altered miRNAs. **f** Heatmap of two differentially expressed miRNAs in plasma-derived EVs displays log2-transformed expression values, with red indicating high expression and blue indicating low expression (fold change ≥2, FDR *P* < 0.05).

On postoperative day 3, CSF samples were collected (Fig. 1a); the number of red blood cells in each sample was <1 (Supplementary Table 1). EVs isolated from both CSF and plasma were characterized by NanoFCM, with particle sizes ranging from 50 to 200 nm and a predominant peak at approximately 70–80 nm (Supplementary Fig. 1). Transmission electron microscopy (TEM) further revealed particles with a lipid bilayer membrane (Supplementary Fig. 1). Fluorescently labeled EVs were analyzed by NanoFCM, with phosphate-buffered saline (PBS) processed identically as a negative control. Signals for CD81 (approximately sixfold), ALIX (above the detection threshold), and TSG101 (approximately 28-fold) were elevated relative to the control (Supplementary Table 2), exceeding the reported NanoFCM measurement error (10–30%) and thereby confirming specific marker detection^31^. The concordant elevation of all three markers supports the presence of fluorescently labeled EVs, notwithstanding validation in a single sham-derived sample (n = 1). The particle size distribution falls within the expected EV range (Supplementary Fig. 2). Because identical isolation procedures were applied to SCI and sham cerebrospinal fluid and plasma samples, this single-sample validation was considered sufficient to verify methodological integrity. These findings demonstrate the successful isolation and purification of EVs from both the CSF and plasma.

### miR-9a-3p upregulation in CSF-EVs following SCI in rats

After quality control, 22 samples (CSF SCI, n = 5; CSF sham, n = 6; plasma SCI, n = 5; plasma sham, n = 6) were included in the final analysis (Supplementary Table 3). To evaluate potential batch effects, principal component analysis (PCA) was performed (Supplementary Fig. 3), and the PC1 and PC2 scores were compared between the sequencing batches (first vs. second) using Welch’s t-test. No significant differences were observed (PC1: *P* = 0.704; PC2: *P* = 0.970), indicating minimal batch effects. Additionally, permutational multivariate analysis of variance (PERMANOVA) confirmed that batch effects were negligible across the entire PCA space (R² = 0.0048, *P* = 0.879). Thus, all samples were analyzed without batch correction.

PCA of miRNA sequencing data from CSF-derived EVs revealed distinct clustering between the sham and SCI groups, whereas plasma samples showed minimal differences (Fig. 1b). Among the CSF-EV miRNAs, 42 were differentially expressed, most notably miR-9a-3p (fold change = 11.66, false discovery rate [FDR] *P* = 1.83 × 10^−17^) (Fig. 1c, d). Several other miRNAs such as miR-21-5p and miR-124-3p also showed increased expression in the SCI group, while miR-204-5p was significantly downregulated. Although they were not analyzed further in the present study, these miRNAs have been previously associated with neuroinflammation or neuroprotection^32–34^. In plasma, only two miRNAs met the differential expression criteria, including miR-9a-3p (fold change = 7.91, FDR *P* = 6.29 × 10^−4^) (Fig. 1e, f). These results suggested that CSF-derived EVs reflected SCI-induced miRNA alterations more sensitively than plasma-derived EVs. Quantitative polymerase chain reaction (qPCR) validated the sequencing findings by confirming significantly higher miR-9a-3p expression in CSF-derived EVs from SCI rats than in sham controls (*P* = 3.80 × 10^−3^) (Supplementary Fig. 4), indicating that increased miR-9a-3p levels were robust and reproducible.

### miR-9a-3p is highly expressed in astrocytes of the brain

**Fig. 2:**
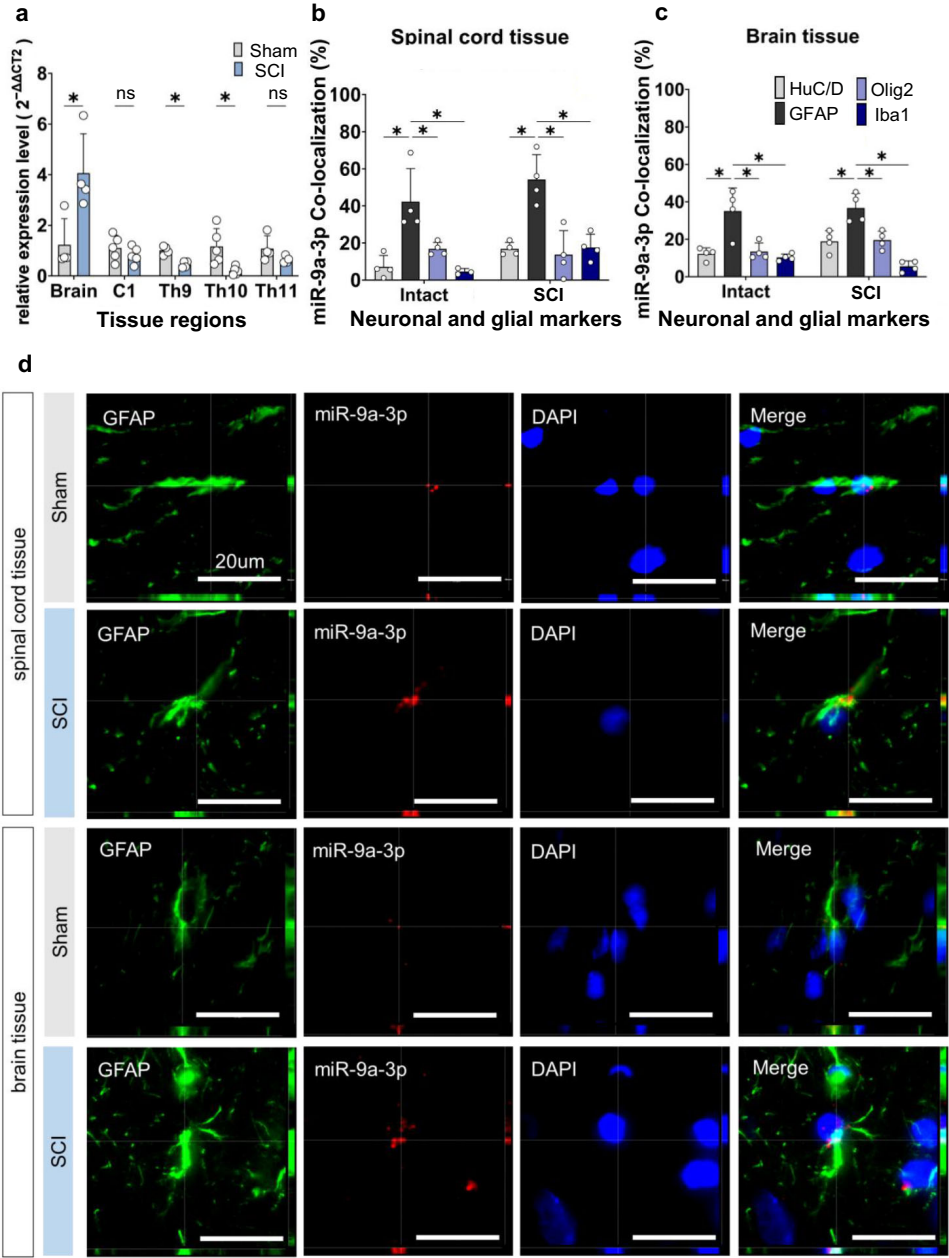
miR-9a-3p expression analysis in the spinal cord and brain of rats. **a** qPCR analysis of miR-9a-3p expression in the spinal cord and brain (primary motor cortex). In the SCI model, miR-9a-3p expression was significantly decreased at Th10 (sham vs. SCI, *P* = 0.017) and Th9 (sham vs. SCI, *P* = 0.0019), whereas a significant increase was observed in the brain (sham vs. SCI, *P* = 0.023). *P < 0.05, ns not significant; two-sided unpaired Student’s *t* test. Sample sizes were as follows: brain, n = 4; C1, n = 5; Th9, n = 4; Th10, n = 5; Th11, n = 4. **b** Quantification of miR-9a-3p expression in the spinal cord. Astrocytes exhibited significantly higher miR-9a-3p expression than neurons, oligodendrocytes, and microglia (*P* < 0.001, two-way ANOVA, column factor). Intergroup comparison between sham and SCI spinal cords was not performed to avoid potential bias due to variations in slide conditions. Sample sizes were n = 4 in each group. **c** Quantification of miR-9a-3p expression in the brain. Astrocytes showed significantly higher miR-9a-3p expression than neurons, oligodendrocytes, and microglia (*P* < 0.001, two-way ANOVA, column factor). Intergroup comparison between the sham and SCI groups was not performed to ensure consistency in slide preparation. Sample sizes were n = 4 in each group. **d** FISH and immunostaining of miR-9a-3p in astrocytes from the spinal cord and brain. Z-stack analysis confirmed miR-9a-3p colocalization with astrocyte markers, with signals observed in both cytoplasm and nucleus. Sample sizes were n = 4 in each group. Error bars represent mean ± SD.

To investigate the potential cellular origin of EVs, we performed qPCR for miR-9a-3p across the brain (primary motor cortex) and C1, Th9, Th10 (lesion site), and Th11 spinal cord levels in both sham and SCI rats (brain, n = 4; C1, n = 5; Th9, n = 4; Th10, n = 5; Th11, n = 4). SCI rats exhibited significantly lower miR-9a-3p levels at the lesion site (Th10; *P* = 0.017 vs. sham) and proximal level (Th9; *P* = 1.90 × 10^−3^ vs. sham) but significantly higher expression in the brain (primary motor cortex; *P* = 0.023 vs. sham). No notable differences were observed at the C1 and Th11 level (Fig. 2a).

Next, we performed Fluorescent in situ hybridization (FISH) of neurons, astrocytes, oligodendrocytes, and microglia to localize miR-9a-3p in both the spinal cord and brain of sham and SCI animals (Supplementary Figs. 5 and 6). Quantitative analysis revealed that miR-9a-3p expression was significantly higher in astrocytes than in neurons, oligodendrocytes, and microglia (*P* < 0.001, two-way analysis of variance [ANOVA], column factor) (Fig. 2b, c). miR-9a-3p expression in astrocytes was confirmed in both the spinal cord and brain (Fig. 2d). All FISH staining was performed under identical conditions. However, due to minor variability in staining intensity across slides, direct quantitative comparisons between sham and SCI animals were not conducted. Quantitative analyses were limited to comparisons among different cell types within each group, based on internally controlled measurements.

### miR-9-3p alterations in human SCI CSF samples

In a previous clinical trial^35^, CSF samples were collected from patients with SCI. All participants had complete paralysis (modified Frankel A) 72 h post-injury. They were subsequently categorized into two groups: the non-recovery group, consisting of patients who remained in modified Frankel A on day 168, and the recovery group, comprising individuals who showed spontaneous improvement to modified Frankel B or C by day 168 (Supplementary Table 4). For comparative analysis, CSF samples were obtained from individuals with uninjured spinal cords. Ultimately, 18 samples (control, n = 4; non-recovery, n = 9; recovery, n = 5) were analyzed (Supplementary Table 5). The baseline characteristics of the study participants are presented in Table 1. No significant differences were observed between the groups with respect to age (ANOVA, *F*(2,15) = 1.80, *P* = 0.20) or sex (Fisher’s exact test, *P* = 0.255). Although the sample size was small, these findings suggest minimal demographic bias.

**Table 1:** Comparison of demographic and clinical characteristics among the control, non-recovery, and recovery groups

Variable	Control (n = 4)	Non-recovery (n = 9)	Recovery (n = 5)	Fisher’s exact test
Age (years)	53.8 ± 3.3	55.4 ± 20.2	63.0 ± 7.4	*F* (2,15) = 1.80, *P* = 0.20
Sex (male), %	50.0	77.8	100.0	*P* = 0.255
Race (East Asians), %	100.0	100.0	100.0	N/A
Modified Frankel grade at baseline, %				N/A
A	N/A	100.0	100.0	
B	N/A	0	0	
C	N/A	0	0	
Modified Frankel grade at follow-up, %				*P* = 0.0079 (control vs. recovery), *P* = 0.0005 (non-recovery vs. recovery)
A	N/A	100	0	
B	N/A	0	80.0	
C	N/A	0	20.0	

Values are presented as mean ± standard deviation for continuous variables and n (%) for categorical variables. The group sizes were as follows: control (n = 4), non-recovery (n = 9), and recovery (n = 5). Age (analysis of variance, *F* (2,15) = 1.80, *P* = 0.20) and sex (Fisher’s exact test, *P* = 0.255) were not significantly different among the control, non-recovery, and recovery groups. The modified Frankel grade at follow-up was significantly different between the control and recovery (Fisher’s exact test, *P* = 0.0079) and between the non-recovery and recovery groups (*P* = 0.0005).

N/A, the variable is not applicable to the control group.

**Fig. 3:**
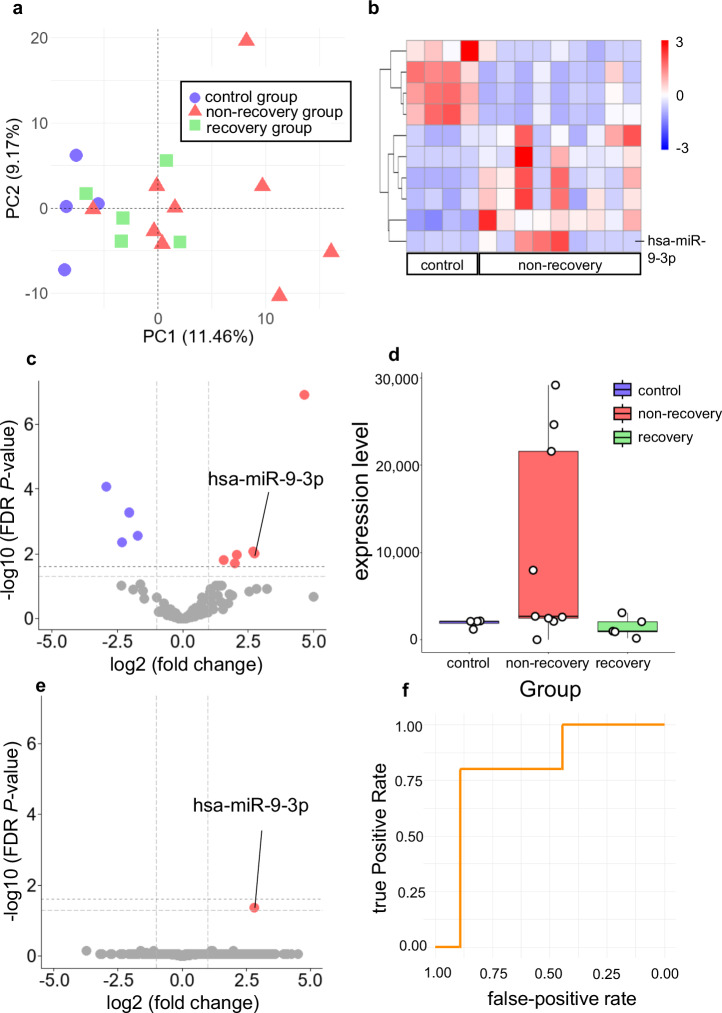
Differential miRNA expression analysis in CSF-derived EVs from human patients with SCI. **a** PCA score plot of CSF samples from the control (n = 4), non-recovery (n = 9), and recovery (n = 5) groups. Each dot represents an independent sample, showing distinct clustering among the three groups (blue circles, control; red triangles, non-recovery; green squares, recovery). **b** Heatmap of 10 differentially expressed miRNAs between the control and non-recovery groups in CSF-derived EVs. Colors represent log2-transformed expression values, with red indicating high expression and blue indicating low expression (fold change ≥2, FDR *P* < 0.05). **c** Volcano plot of differentially expressed miRNAs between the control and non-recovery groups in CSF-derived EVs. The x-axis indicates log2 fold change, and the y-axis represents −log10-transformed FDR *P* value. Red dots represent significantly upregulated miRNAs (fold change ≥2 and FDR *P* < 0.05), while blue dots represent significantly downregulated miRNAs (fold change ≤−2 and FDR *P* < 0.05). miR-9-3p is highlighted among the significantly altered miRNAs. The comparison between the non-recovery and recovery groups for miR-9-3p yielded an adjusted FDR *P* = 0.0189. **d** Box plot of miR-9-3p expression in CSF-derived EVs among control, non-recovery, and recovery groups. miR-9-3p expression was significantly different between the control and non-recovery groups (adjusted FDR *P* = 0.0189). The difference between the recovery and non-recovery groups showed a trend (adjusted FDR *P* = 0.08). **e** Volcano plot of differentially expressed miRNAs between the recovery and non-recovery groups in CSF-derived EVs. The x-axis indicates log2 fold change, and the y-axis represents −log10-transformed FDR *P* value. Red dots represent significantly upregulated miRNAs (fold change ≥2 and FDR *P* < 0.05). miR-9-3p is among the significantly altered miRNAs, with an adjusted FDR *P* = 0.08 for comparison between the recovery and non-recovery groups. **f** Receiver operating characteristic curve analysis for miR-9-3p in CSF-derived EVs from SCI patients. The AUC was 0.80, with a cutoff of 2097.92 CPM, sensitivity of 80%, and specificity of 89%.

The PCA of the miRNA sequencing data demonstrated clear clustering among the control, non-recovery, and recovery groups (Fig. 3a). Relative to the non-recovery group, 10 miRNAs, including miR-9-3p, were significantly altered in the control group (fold change ≥2, FDR *P* < 0.05) (Fig. 3b, c). Notably, miR-9-3p (the human ortholog of rat miR-9a-3p) had an FDR *P* = 0.0094 (corrected *P* = 0.019) (Fig. 3d). When comparing the non-recovery and recovery groups, miR-9-3p was the only miRNA displaying a significant change in expression (FDR *P* = 0.04), although the corrected FDR *P* value (0.08) was slightly above 0.05 (Fig. 3d, e). Furthermore, receiver operating characteristic curve analysis (Fig. 3f) demonstrated that miR-9-3p exhibited strong predictive power for recovery, with an area under the curve (AUC) of 0.80. The optimal cutoff value was identified as 2097.92 counts per million (CPM), providing a sensitivity of 80% and a specificity of 89%. Collectively, these results suggest that miR-9-3p is specifically regulated in SCI and is a promising biomarker for predicting recovery. Within the non-recovery group, three patients (PR434207a, PR434209a, PR434210a) exhibited relatively high miR-9-3p expression. These patients sustained traumatic cervical SCI due to high-energy falls, had modified Frankel grade A at baseline, and underwent posterior fixation surgery (Supplementary Table 4). CSF was collected prior to surgery in all three cases. No obvious clinical differences such as age, neurological level, or injury mechanism were noted when compared to the remaining patients in the non-recovery group (Supplementary Table 4).

### miR-9-3p suppresses energy metabolism and induces gene programs suggestive of neuroprotective responses

**Fig. 4:**
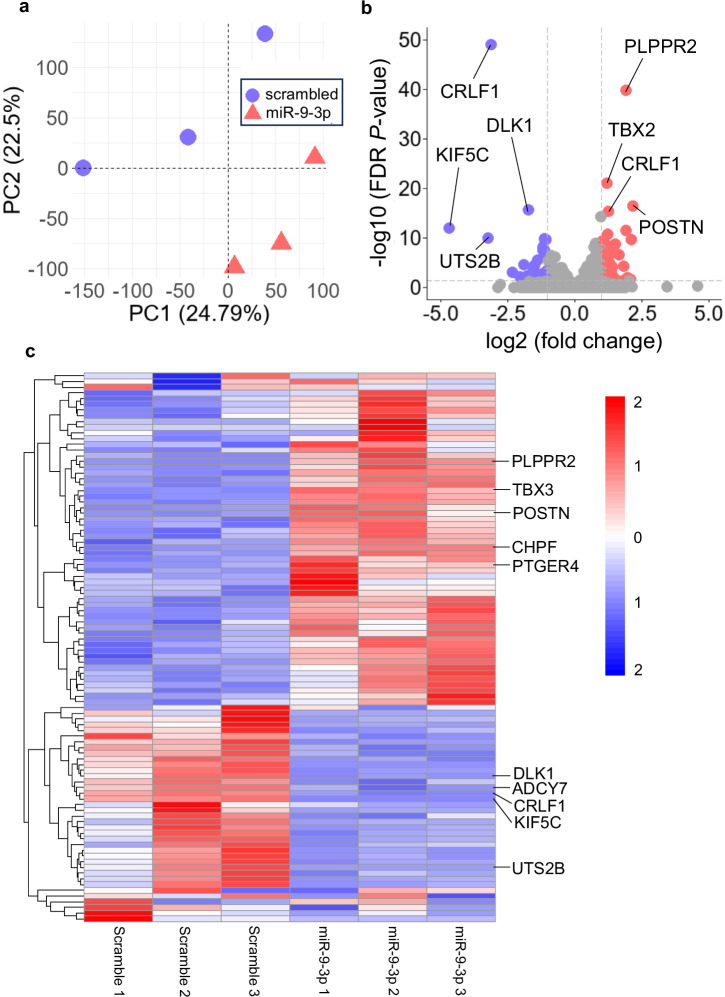
Transcriptomic analysis of human motor neurons transduced with miR-9-3p. **a** PCA score plot of the scramble (n = 3) and miR-9-3p (n = 3) groups. Each dot represents an independent sample, illustrating distinct clustering between the two groups (blue circles, scramble; red triangles, miR-9-3p). **b** Volcano plot of differentially expressed RNAs between the scramble and miR-9-3p groups. The x-axis indicates log2 fold change, and the y-axis represents −log10-transformed FDR *P* value. Red dots (n = 34) represent significantly upregulated RNAs (fold change ≥2, FDR *P* < 0.05), whereas blue dots (n = 53) represent significantly downregulated RNAs under the same criteria. **c** Heatmap of 87 differentially expressed RNAs between the scramble and miR-9-3p groups. Colors represent log2-transformed expression values, with red indicating high expression and blue indicating low expression (fold change ≥2, FDR *P* < 0.05).

To elucidate the potential mechanism, we overexpressed miR-9-3p in human-derived motor neurons and performed RNA sequencing. qPCR confirmed significantly elevated miR-9-3p levels in the overexpression group compared with those in the scramble controls (*P* = 0.025) (Supplementary Fig. 7), and PCA of the transcriptomic data demonstrated a clear separation between the two groups (Fig. 4a).

**Fig. 5:**
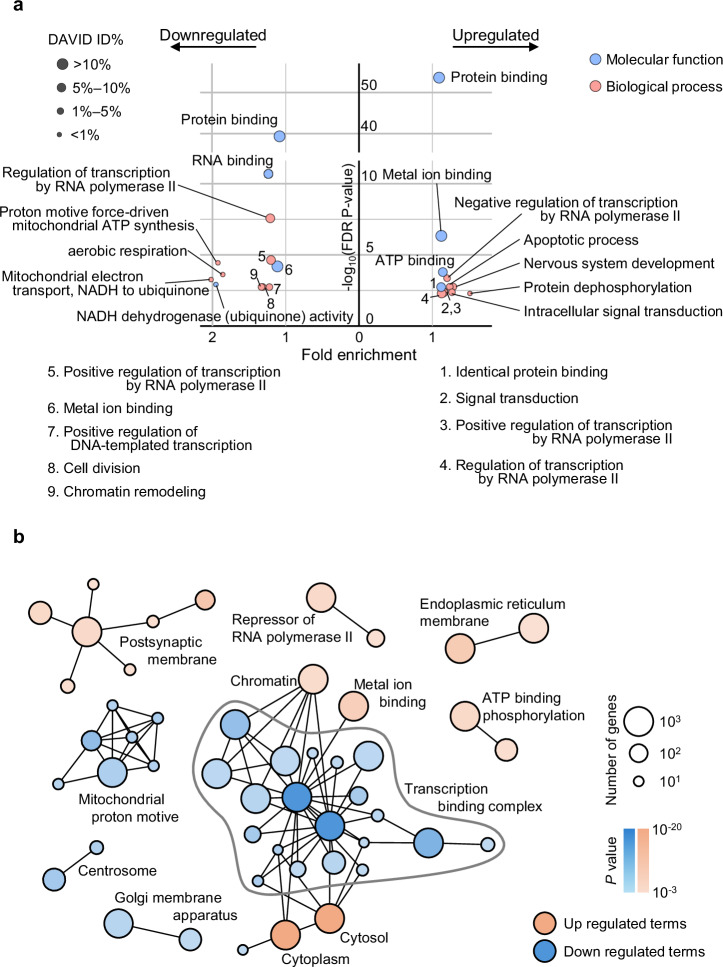
Summary of the GO enrichment analysis and functional network. **a** Dot plots of significantly enriched GO terms in the biological process (BP) and molecular function (MF). The dot size represents the fold change between observed and expected counts. A total of 8 suppressed BP terms and 4 suppressed MF terms, as well as 8 upregulated BP terms and 4 upregulated MF terms, are shown (FDR *P* < 0.05). Some GO terms, such as ‘regulation of transcription by RNA polymerase II,’ appeared in both upregulated and downregulated categories, reflecting the presence of functionally divergent genes within the same annotation. **b** Network analysis of differentially expressed genes identified in this study. Functional clustering indicates that miR-9-3p modulates biological pathways by inhibiting energy metabolism and promoting gene programs associated with neuroprotective responses.

Using fold change ≥2 and FDR *P* < 0.05 as cutoffs, we identified 34 downregulated and 53 upregulated transcripts (Fig. 4b, c). Gene Ontology (GO) analysis using Database for Annotation, Visualization, and Integrated Discovery (DAVID) revealed enriched terms related to transcription/translation repression, cell cycle arrest, and suppression of energy metabolism among the downregulated genes, whereas the upregulated genes were associated with nervous system development and function (Fig. 5a). It is worth noting that some GO terms, such as ‘regulation of transcription by RNA polymerase II,’ appeared in both upregulated and downregulated categories. This reflects the inclusion of distinct gene subsets with divergent directions of change within the same GO term, likely reflecting complex regulatory dynamics rather than a simple unidirectional effect.

GO network analysis revealed distinct clusters associated with gene programs implicated in neuroprotective responses and energy metabolism-related processes. Post-synaptic membrane-related terms were upregulated, suggesting that miR-9-3p contributes to cellular programs associated with neuroprotective responses, whereas the increased expression of transcriptional repressors and reduced activity of transcription complexes or the Golgi apparatus implied lower overall transcription and energy production. The upregulation endoplasmic reticulum membrane–membrane-associated terms further suggested an enhanced response to damaged proteins (Fig. 5b).

**Fig. 6:**
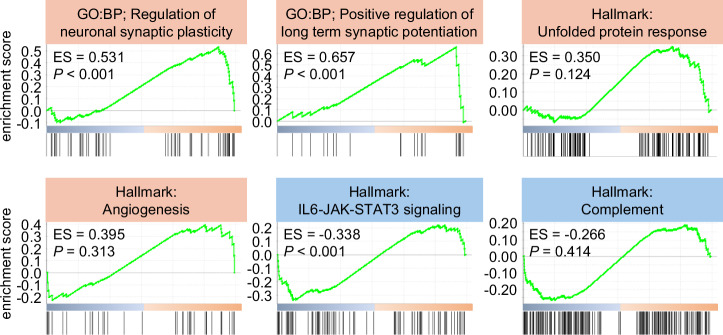
GSEA analysis of miR-9-3p modulation. GSEA enrichment plots of representative gene sets (*P* < 0.05). The top-ranking gene sets are displayed, illustrating the key biological pathways influenced by miR-9-3p expression.

Gene set enrichment analysis (GSEA) showed negative enrichment for gene sets associated with energy metabolism (e.g., oxidative phosphorylation, mitochondrial proton motive force) but positive enrichment for synaptic plasticity (e.g., regulation of neuronal synaptic plasticity, positive regulation of long-term synaptic potentiation) and protein-damage response (e.g., unfolded protein response) (Fig. 6). Collectively, these data indicated that miR-9-3p exerts multifaceted effects on energy metabolism, immune regulation, and oxidative stress pathways in SCI, potentially contributing to injury adaptation and supporting functional recovery.

## Discussion

**Fig. 7:**
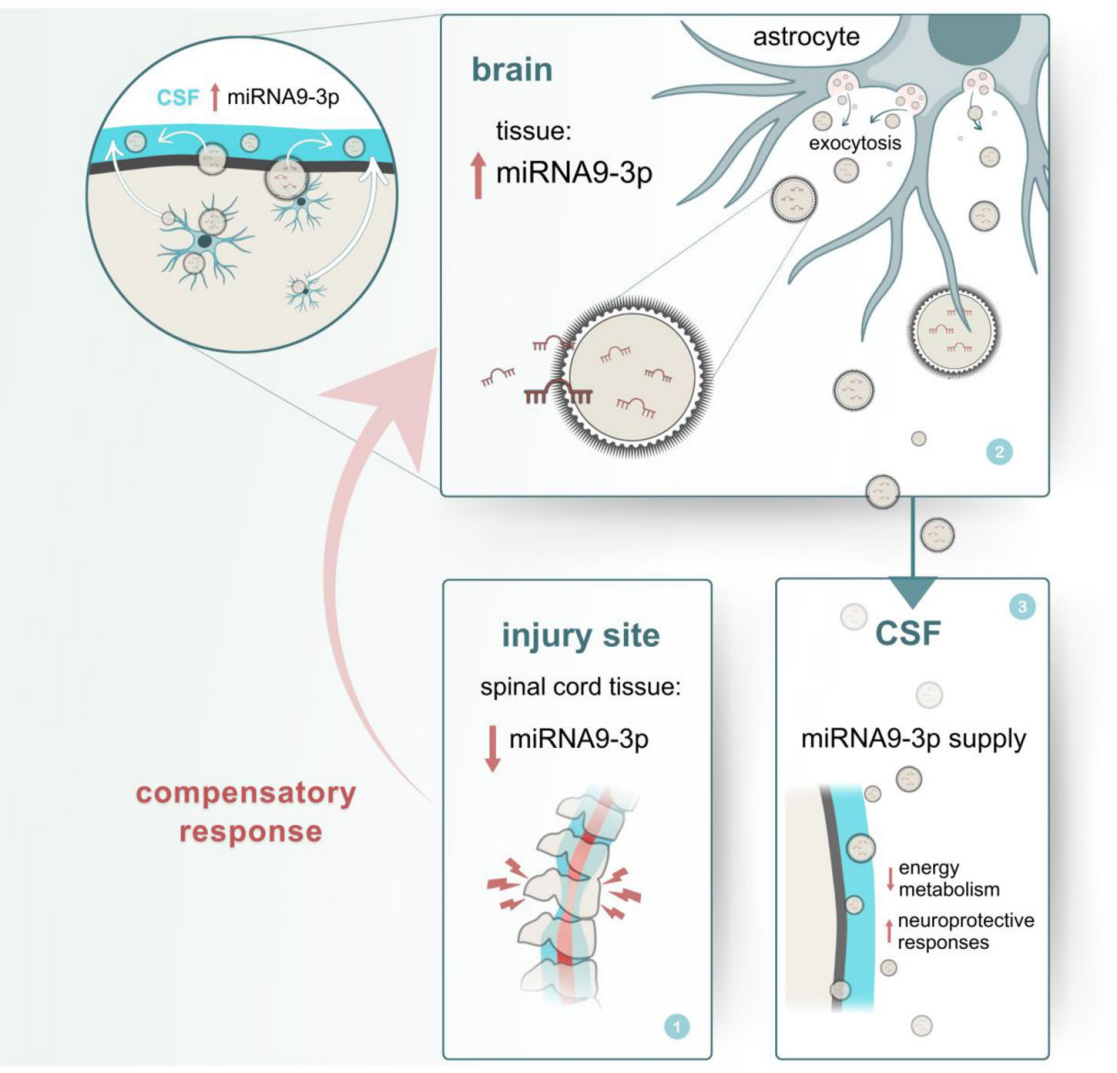
Proposed schematic illustrating miR-9-3p dynamics in acute SCI. During the acute phase of SCI, miR-9-3p is released via EVs and potentially contributes to the suppression of energy metabolism and neuroprotective responses. Illustration created by Ivana Duic Urushibata.

In the present study, we focused on CSF-derived EV miRNAs to elucidate the molecular mechanisms underlying acute SCI. Although previous studies have reported the presence of miR-9-3p in the CSF of SCI patients and suggested its potential involvement in SCI pathology^36^, the specific functional roles and the prognostic value of EV-encapsulated miR-9-3p in spontaneous recovery remain to be elucidated and are addressed for the first time, to the best of our knowledge, in our study. We observed a specific change in miR-9-3p levels within CSF-derived EVs in both rat model and human samples. Notably, analyses using a rat model demonstrated that miR-9-3p expression decreased at the lesion site but increased in the brain after SCI. The miR-9-3p is highly expressed in brain astrocytes, raising the possibility that astrocyte-derived EVs contribute to its elevated levels in the CSF. Functional analyses revealed that miR-9-3p suppresses energy metabolism and induces transcriptomic changes associated with stress responses and synaptic function. These changes suggest neuroprotective implications, such as metabolic downregulation and structural stabilization, rather than direct inhibition of apoptosis. Elevated levels of miR-9-3p in CSF-derived EVs during the acute phase of SCI indicate a potential role in mitigating secondary damage and supporting the maintenance of structural integrity and functional recovery (Fig. 7). This supports the idea that miR-9-3p contributes to central nervous system (CNS) resilience after SCI through EV-mediated inter-CNS communication. CNS resilience likely involves multiple biological processes. Among them, neuroprotection refers to the preservation of existing neural tissue, while neuroplasticity involves the reorganization of neural networks to compensate for injury. Our findings suggest that miR-9-3p contributes to both processes through distinct mechanisms, although further in vivo validation is required.

Our findings in human samples indicated that CSF-derived EV miR-9-3p is a viable biomarker for predicting spontaneous recovery. In our cohort, all patients were classified as modified Frankel A 72 h post-injury. This observation is particularly important because the American Spinal Injury Association Impairment Scale (AIS) assessments at 72 h are more reliable for determining the severity of paralysis than those conducted within 24 h, which can be confounded by factors such as spinal shock, medical instability, and altered consciousness^37^. A previous study reported that free circulating miR-9-3p in the CSF correlates with AIS scores at 24 h^36^; however, sample collection was performed within 24 h post-injury, which reduces the reliability of the findings for broader application. Therefore, our findings demonstrate the relevance of this biomarker at later time points. Furthermore, while their study identified miR-9-3p as a potential marker for distinguishing the severity of paralysis in the acute phase, our study highlighted its potential as a longitudinal marker for spontaneous recovery, further underscoring its clinical significance. To the best of our knowledge, this is the first study to identify an EV-associated miRNA—specifically, miR-9-3p—as a biomarker predictive of spontaneous neurological recovery after SCI. This novelty lies not only in the focus on spontaneous recovery but also in the use of CSF-derived EVs, which likely reflect CNS-specific pathophysiological processes more directly than plasma-derived EVs. Importantly, encapsulation within EVs further enhances the clinical utility of miR-9-3p by increasing its stability and reducing variability associated with free-circulating forms. Indeed, miRNAs contained in EVs typically exhibit heightened resilience, providing increased protection against degradation^38^. In contrast, previous studies have reported that miR-9-3p in its free-circulating form is inherently more vulnerable and prone to variability stemming from pre-analytical factors^39,40^. Given these differences, our identification of miR-9-3p in CSF-derived EVs further enhances its potential as a robust biomarker. Although we cannot completely rule out the possibility that miR-9-3p levels partly reflect subtle differences in injury severity even within the modified Frankel A classification, the significant expression differences observed between the recovery and non-recovery groups—despite identical baseline neurological status—suggest that miR-9-3p is also indicative of the intrinsic capacity for spontaneous recovery. Furthermore, within the non-recovery group, a distinct subset of patients exhibited substantially elevated miR-9-3p expression. Despite sharing similar clinical characteristics—including modified Frankel grade, neurological injury level, and timing of CSF collection—these individuals displayed markedly divergent miRNA expression profiles. This divergence indicates that miR-9-3p expression is influenced by inter-individual biological responses to SCI, independent of injury severity. Such variability underscores the utility of miR-9-3p as a molecular indicator of intrinsic recovery potential, providing insights beyond conventional clinical classification. This raises an important question: do some patients recover simply because of imperceptible differences in initial severity, or are there molecular mechanisms that actively promote recovery? While this study cannot fully resolve that question, the observed expression differences in patients with the same clinical severity (modified Frankel A) support the hypothesis that miR-9-3p is associated with recovery potential.

We summarized the previous reports on biomarkers that reflect the degree of natural recovery after SCI in human samples in Supplementary Table 6. Studies reporting biomarkers for natural recovery using human samples are extremely limited, with only 10 studies included in the present study. Although previous studies have explored EV miRNAs in plasma following SCI^41^, this is the first study to establish a specific EV-associated biomarker—miR-9-3p—that predicts spontaneous recovery, based on both animal models and clinical CSF samples. Among the reported biomarkers, EV-associated miR-9-3p demonstrates remarkable features for predicting natural recovery after SCI. As reported in the Results section and shown in Fig. 3f, miR-9-3p in CSF-derived EVs exhibited high predictive accuracy for long-term outcomes at 2 years post-injury (AUC = 0.80, sensitivity = 80%, specificity = 89%). Although the sample sizes and study conditions differed, previously reported biomarkers such as CSF neurofilament light (AUC, 0.89; sensitivity, 81%; specificity, 79%) and CSF glial fibrillary acidic protein (GFAP) (AUC, 0.87; sensitivity, 78%; specificity, 80%) demonstrated high predictive value at 6 months post-injury^42^. While direct comparison between these biomarkers is not possible due to differences in cohorts, methodologies, and time points, our findings highlight the potential of miR-9-3p as a robust and long-term prognostic marker. Moreover, unlike protein biomarkers, miRNAs can be amplified via PCR, allowing for highly sensitive detection even with minimal sample volumes. This property supports the utility of miR-9-3p as a sensitive and feasible biomarker, especially when only small volumes of CSF can be obtained. Furthermore, unlike predictive models that rely on combinations of multiple proteins analyzed using machine learning, miR-9-3p achieves high predictive accuracy as a single biomarker. Given that many studies have improved prediction accuracy by combining multiple biomarkers, it is exceptionally rare for a single molecule to exhibit such a strong predictive ability. These findings highlight the clinical utility and simplicity of miR-9-3p as a biomarker.

Regarding the source of secreted miR-9-3p in CSF-derived EVs, two possibilities merit consideration: (1) active release from the CNS and (2) influx from the bloodstream. Considering the latter possibility, the blood–spinal cord barrier is disrupted within minutes of acute SCI^43–45^ and reaches maximal permeability around postoperative day 3^45^. Concurrently, extensive hemorrhage is already present at this time and persists until 5 days post-injury in the spinal cord tissue^46^. Given both the increased vascular permeability and the presence of hemorrhage, blood contamination in the CSF cannot be completely ruled out. However, our data suggest that such contamination is minimal, as miR-9-3p levels in the plasma remain extremely low (Supplementary Fig. 8). This finding supports the notion that the majority of miR-9-3p in CSF-derived EVs originate in the CNS.

The next question was which regions of the CNS secrete miR-9-3p. Under intact conditions, the CNS contains abundant miR-9-3p^47,48^ However, upon injury, our data showed that miR-9-3p levels increased markedly in the brain during the acute phase of SCI, whereas they decreased in both the lesion site and proximal regions. A plausible explanation is that the brain, which exhibits elevated miR-9-3p expression, releases EVs containing miR-9-3p into the CSF. Indeed, it has been demonstrated that when a particular miRNA is highly enriched in EVs, it often originates from the tissues where it is most abundant^49,50^. Thus, it is likely that brain-derived miR-9-3p actively contributes to the pathology of SCI.

In contrast, how can we explain this downregulation at the injury site? Several factors appear to contribute to this phenomenon. One key mechanism involves transcriptional regulators that suppress specific miRNAs^6,51^. Among these, the RE1-silencing transcription factor (REST) has been suggested to bind directly to miR-9-3p^52–54^, thereby downregulating its expression. However, its precise role remains unclear, necessitating further investigation, particularly using REST overexpression models, to elucidate its regulatory functions in this context. Another contributing factor is the altered cellular composition at the lesion site following SCI^48^. During the acute post-injury phase, a reduction in the astrocyte population has been reported^55^, which could contribute to decreased levels of miR-9-3p at the injury site. Collectively, these mechanisms provide a plausible explanation for the downregulation of miR-9-3p expression after SCI.

Elevated miR-9-3p levels in the brain are potentially associated with central plasticity, which facilitates functional recovery after SCI. One possible contributor to this plasticity is high-frequency gamma-wave transmission from the nucleus accumbens to the primary motor cortex (M1) during the acute phase of SCI^56^. This process is linked to the induction of miRNAs involved in stress adaptation. In support of this idea, certain miRNAs with reported neuroprotective roles are upregulated in the brain following SCI^57^. Our findings demonstrated the upregulation of miR-9-3p in the brain, consistent with a role in injury-associated plasticity. Consistent with these observations, previous studies reported that miR-9-3p promotes neural progenitor cell differentiation^47,52,58,59^. Within the brain, miR-9-3p is predominantly localized in astrocytes, as shown in this study, and is largely assumed to be released from astrocytes following SCI. This is supported by the fact that 63% of the miRNAs detected in induced pluripotent cells-derived astrocyte EVs reported in a previous study were also detected in the CSF EVs in this study^60^. Other studies have shown that astrocyte-derived EVs contribute to protective mechanisms in neurological disorders^61–64^. Based on these observations, it is plausible that miR-9-3p is upregulated in astrocytes, released into the CSF, and subsequently exerts beneficial effects on the injured spinal cord. Indeed, in our human samples, higher miR-9-3p levels in CSF-derived EVs were detected in individuals who exhibited spontaneous recovery, suggesting a protective role for miR-9-3p in the repair process.

Our forced expression experiments in human motor neurons showed that miR-9-3p downregulates energy metabolism and induces gene expression changes associated with cellular stress adaptation and neuronal function, which are indicative of neuroprotective implications at the transcriptional level. During the acute phase of SCI, a transient suppression of energy metabolism or mitochondrial function has the potential to be linked to an adaptive, protective strategy against cell death^65,66^. Our findings support the involvement of miR-9-3p in facilitating such a torpor-like state, previously linked to reduced metabolic stress and improved neuronal viability^65,66^.

Our study has several limitations. First, the animal model did not fully replicate human SCI pathology. The severity of SCI differs among species, making direct comparisons difficult. Second, the sample size was limited. Although the study was conducted after a randomized controlled trial involving multiple centers, additional cases are needed for stronger conclusions. Third, direct evidence of miR-9-3p function in the injured spinal cord is lacking, and its release from astrocytes remains unconfirmed. Furthermore, the mechanism by which miR-9-3p is incorporated into extracellular vesicles also requires further investigation. These limitations necessitate cautious interpretation of the results. Fourth, the study examined only a single acute time point, limiting assessment of temporal changes in miR-9-3p expression. Future studies including multiple time points are needed to clarify its dynamic role in SCI progression.

In conclusion, although miR-9-3p has traditionally been viewed as a “passenger strand”^36,67^, its guide strand counterpart miR-9-5p has been well studied and is known to regulate neurodevelopment, plasticity, and inflammation following SCI. In contrast, functional roles of miR-9-3p remain largely unexplored. Given that miR-9-5p and miR-9-3p strands are processed from the same precursor miRNA but have different sequences (miR-9-3p: AUAAAGCUAGAUAACCGAAAGU; miR-9-5p: UCUUUGGUUAUCUAGCUGUAUGA), they are expected to target distinct sets of RNAs and exhibit different cellular localization and functions, as previously shown in other miRNA pairs^68,69^. Therefore, mechanistic analysis of miR-9-3p should be conducted independently of miR-9-5p. Our findings suggest that it plays a critical role in the endogenous protective and adaptive response of the CNS to SCI. Furthermore, CSF-derived EV miR-9-3p shows promise as a sensitive biomarker for diagnosing SCI and evaluating therapeutic efficacy. Future research needs to focus on molecular therapeutics targeting miR-9-3p and large-scale prospective clinical studies to validate its potential as a biomarker and ultimately improve outcomes in patients with SCI.

## Methods

### Study design

This study aimed to investigate the physiological role of EV-derived miR-9-3p in acute SCI and evaluate its potential as a biomarker for predicting recovery (ClinicalTrials.gov identifier, NCT02193334). We analyzed miR-9-3p expression, cellular origin, and functional impact in a rat SCI model and human CSF samples. The sample size was determined based on expert judgment, informed by prior experimental data and practical feasibility. The exact n values for each experiment are listed in the figure legends. The experimental unit was a single rat. Randomization was not formally performed; animals were assigned to experimental groups without any intentional selection or systematic allocation. For the in vivo experiments, animals that died or experienced severe health complications were excluded in accordance with ethical guidelines. Animal and sample allocation as well as data acquisition (in vivo and ex vivo) were conducted in a blinded manner; however, the investigators were not blinded during data analysis because of study design constraints. Outliers were defined based on the statistical criteria before the study began. For miRNA sequencing of rat CSF and plasma samples, outliers were defined as those with miRNA concentrations below 20 pg/µL to ensure sufficient RNA input for sequencing. In human CSF sample analysis, outliers were identified using robust PCA (RPCA) based on Mahalanobis distances and an interquartile range (IQR)-based threshold (median + 1.5 × IQR), with excluded samples reported in Supplementary Table 5. These predefined criteria ensured that all analyzed samples met the quality control standards and that outlier handling was transparently reported. No protocol was registered prior to the study. This study adheres to the ARRIVE guidelines for reporting animal research. A completed ARRIVE checklist is provided as a separate file, and all relevant details are reported in the Methods.

### Animal models and creation of SCI

All animal experiments were conducted at Keio University and the Central Institute of Experimental Animals in accordance with the Guidelines for the Care and Use of Laboratory Animals of Keio University (approval no. 13020). Eight-week-old female Sprague–Dawley rats weighing 150–200 g (Sankyo Labo Service Corporation, Inc., Tokyo, Japan) were used. The animals were housed under a 12-h light/dark cycle at 22–24 °C and 40–60% humidity, with free access to food and water. They were acclimated for 1 week prior to surgery, and their food and water intake was monitored daily. The housing environment was cleaned regularly and inspected to maintain animal health.

Anesthesia was induced via subcutaneous injection into the dorsal region of a mixture of midazolam (0.15 mg/kg), medetomidine (0.075 mg/kg), and butorphanol (0.25 mg/kg), diluted in saline. A volume of 0.1 mL was administered, and the injection site was disinfected with 70% ethanol prior to administration.

In the sham model, a laminectomy was performed at the Th10 vertebral level after skin incision and muscle dissection, avoiding direct compression or injury to the spinal cord. In the SCI group, the spinal cord at Th10 was exposed and injured by applying constant pressure for 5 s using modified no. 5 Dumont forceps with a 0.5 mm flat tip. The forceps were equipped with a spring mechanism to ensure consistent pressure across animals. The instruments were cleaned with 70% ethanol and sterilized by autoclaving before use. After confirming the absence of hemorrhage or additional damage, the wound was sutured and closed. This injury protocol was designed to elicit a severe and sustained motor deficit. Given the prolonged compression duration and broader tip geometry, it likely induces damage of equal or greater severity than previously reported compression-based SCI models^70^.

Based on persistent motor deficits (final BBB score < 3) and histological evidence of cavity formation at the injury epicenter, this model was defined as a severe compression SCI model.

After surgery, bladder expression was performed twice daily by gently pressing the abdomen while the rats were in the supine position, until spontaneous urination was observed. To prevent infection, 0.05 mL of the antibiotic Viccure (Sumitomo Dainippon Pharma Animal Health Co., Ltd, Osaka, Japan) was administered subcutaneously on postoperative day 1.

**Fig. 8:**
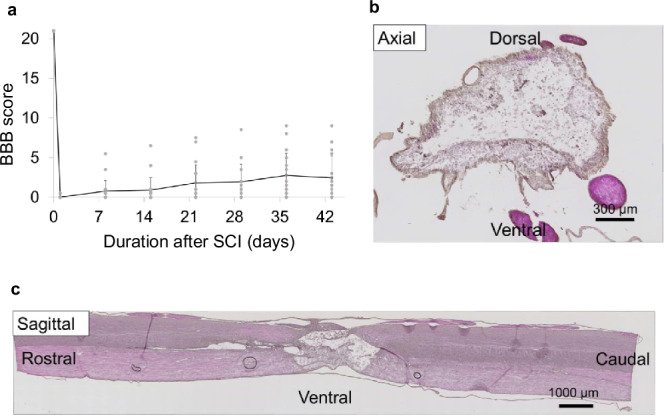
Behavioral and histological assessment of the compression SCI model in rats. **a** Basso, Beattie, and Bresnahan (BBB) scores of rats (n = 20) in a compression SCI model over 42 days post-injury. Scores are presented as mean ± standard error, with a final score of 2.45 ± 2.75. **b**, **c** Histological examination of the injured spinal cord at 42 days post-injury. **b** Representative hematoxylin and eosin-stained cross-sectional view of the injury site, showing prominent cavitation. **c** Longitudinal section of the same injury site, further illustrating extensive tissue loss and cavity formation.

To confirm the successful establishment of the SCI model, behavioral and histological assessments were performed. Locomotor function was evaluated using the Basso, Beattie, and Bresnahan (BBB) scoring system. Rats were monitored for 43 days after surgery, and the final BBB scores averaged 2.45 ± 2.75, indicating persistent motor deficits (Fig. 8a). Hematoxylin and eosin (H&E) staining of spinal cord sections revealed prominent cavity formation at the lesion site (Fig. 8b, c), further supporting the effective induction of SCI. In total, approximately 170 rats were used throughout the study, with different groups allocated for specific analyses: 120 rats for miRNA sequencing, 14 for qPCR validation, 8 for fluorescent in situ hybridization, 20 for behavioral assessment via BBB scoring, 5 for validation of EV surface markers, and 3 for evaluating motor function after CSF sampling. A small number of animals died or were euthanized due to reaching humane endpoints after surgery. However, these instances were rare and did not affect the validity of major statistical analyses. All animals were monitored daily postoperatively, and appropriate care was provided in accordance with predefined welfare criteria. We have complied with all relevant ethical regulations for animal use.

### Collection of CSF and plasma

On postoperative day 3, blood and CSF samples were collected under anesthesia. Day 3 was selected as the earliest feasible time point for CSF collection based on both biological and practical considerations. This timing reflects the acute phase of SCI, during which secondary injury processes such as inflammation and oxidative stress are highly active, but before significant tissue remodeling occurs^71–73^. Earlier than 24–48 h post-injury, rats can be in acute shock and have significant hemorrhage, which could confound CSF sampling. Therefore, postoperative day 3 provided an optimal balance between capturing early pathological signals and ensuring animal well-being. For blood collection, a small incision was made to expose the femoral vein and 1 mL of blood was withdrawn using a 1-mL syringe. The blood sample was transferred to a tube containing ethylenediaminetetraacetic acid and centrifuged at 3000 × *g* for 5 min at 4 °C. Plasma was separated and stored at −80 °C until analysis.

For CSF collection, laminectomy was performed at the L6 vertebral level to expose the dura. The rat was positioned vertically, and a G-1 glass capillary (Narishige, Tokyo, Japan) with a sharp tip (~1.2 cm in length) was inserted through the dura at a ~ 45° angle to withdraw 100 µL of CSF. The collected CSF was stored at −80 °C until further analysis. To assess the potential effects of CSF and blood sampling on motor function, we performed control procedures in three non-injured rats. No decline in BBB score was observed over 14 days of follow-up. All animals subjected to CSF and blood collection were euthanized immediately following sample acquisition. Consequently, these animals were excluded from subsequent functional recovery assessments, including BBB scoring, to avoid potential confounding effects of the sampling procedures on behavioral outcomes.

### EV isolation

CSF or plasma samples were pooled to obtain sufficient volume for EV isolation (1000 µL total for CSF and 500 µL total for plasma, each from 10 animals). The samples were first centrifuged at 1500 × *g* for 20 min at 4 °C, and the supernatant was collected. This supernatant was then centrifuged at 10,000 × *g* for 10 min at 4 °C, and the resulting supernatant was retained.

Concentrated samples were prepared using Amicon Ultra Centrifugal Filter Units (Millipore Sigma): 30 kDa for CSF (to 150 µL) and 50 kDa for plasma (to 150 µL). The concentrated samples (150 µL) were loaded onto qEVsingle columns (Izon, Addington, Christchurch, New Zealand) pre-equilibrated with Dulbecco’s phosphate-buffered saline (DPBS; catalog no. 14249-24; Nacalai Tesque, Kyoto, Japan). After discarding the initial flow-through, EV-containing fractions (fractions 2–5) were eluted in a total volume of 680 µL DPBS. Isolated EVs were stored at 4 °C under sterile conditions until use in the downstream experiments.

### EV characterization

EV size distribution and concentration were measured using a nano analyzer (NanoFCM; NanoFCM Inc., Nottingham, UK). These analyses were performed at the Science and Technology Division of Keio University according to standard protocols. For morphological assessment, EVs were subjected to negative staining and visualized using TEM at the Electron Microscopy Facility, Keio University School of Medicine.

CSF was collected from sham-operated rats (n = 5), with 100 μL obtained from each animal and pooled to yield a total of 500 μL, generating one combined sample for the sham group. The pooled CSF was concentrated to approximately 150 μL using 30 kDa centrifugal filter units (Amicon Ultra-0.5, Merck Millipore). The concentrated CSF (150 μL) was then applied to qEVsingle size-exclusion chromatography columns (Izon, Addington, Christchurch, New Zealand) pre-equilibrated with Dulbecco’s phosphate-buffered saline (DPBS; catalog no. 14249-24; Nacalai Tesque, Kyoto, Japan). After discarding the void volume, extracellular vesicle (EV)-containing fractions (fractions 2–5) were eluted in a total of 680 μL of DPBS. For fluorescent labeling, three 100 μL aliquots of the EV-containing eluate were prepared. DPBS alone served as a negative control. Two of the three aliquots were treated with 0.5 μL of 20% Triton X-100 (Sigma-Aldrich, Triton™ X-100, Cat# X100) and incubated at room temperature for 10 min to permeabilize vesicle membranes. Following membrane permeabilization, 1 μL of fluorescently labeled primary antibody was added to each aliquot, and samples were incubated overnight at 4 °C in the dark. The antibodies used included Alexa Fluor® 488-conjugated anti-Alix [1A12] (Santa Cruz Biotechnology, Cat# SC-53540), Alexa Fluor® 488-conjugated anti-TSG101 [EPR7130(B)] (Abcam, Cat# ab207663), and PE-conjugated anti-mouse/rat CD81 (BioLegend, Cat# 104). Antibodies were diluted 1:50, 1:100, and 1:20, respectively. After incubation, samples were washed using the Vesi-Sec microcolumn system (Meiwafosis, VSEC-35, pore size 35 nm). Columns were pre-spun at 1000 × *g* for 1 min at 15–25 °C. The initial collection tubes were discarded, and the columns were placed into sterile 1.5 mL microcentrifuge tubes. The 100 μL labeled samples were then applied to the columns and centrifuged at 1000 × *g* for 1 min at 15–25 °C using a swing-bucket rotor. The resulting eluates were submitted to Meiwafosis for analysis by NanoFCM. EV size distribution and concentration were measured using a nano analyzer (NanoFCM; NanoFCM Inc., Nottingham, UK), a flow cytometry-based platform optimized for single-particle analysis of nanoscale vesicles. Analyses were performed at the Science and Technology Division of Keio University using standard protocols.

### miRNA sequencing and analysis

Total RNA was extracted from the CSF and plasma samples using the miRNeasy Serum/Plasma Advanced Kit (Qiagen, Hilden, Germany), according to the manufacturer’s instructions. Library preparation and sequencing (Illumina platform) were performed by Takara Bio (Shiga, Japan).

Initially, 24 samples (SCI CSF, n = 6; sham CSF, n = 6; plasma SCI, n = 6; plasma sham, n = 6) were included in the dataset. To ensure data quality, miRNA concentrations were measured using an Agilent 2100 BioAnalyzer, and samples with extremely low miRNA concentrations (<20 pg/µL) were excluded from further analysis. Two samples (SCI CSF, PR399305a; plasma SCI, PR399312a) were removed due to miRNA concentrations of 5.8 and 13.9 pg/µL, respectively, which fell below this predefined quality threshold. After exclusion, 22 samples remained for analysis: SCI CSF, n = 5; sham CSF, n = 6; plasma SCI, n = 5; plasma sham, n = 6. Details of the excluded and retained samples are summarized in Supplementary Table 2.

To minimize technical variability, sequencing was conducted in two separate batches under identical conditions, using the same protocol, reagents, and equipment. The first batch included samples labeled PR3712 (SCI CSF, n = 3; sham CSF, n = 3; plasma SCI, n = 3; plasma sham, n = 3), whereas the second batch (second batch) included samples labeled PR3993 (SCI CSF, n = 3; sham CSF, n = 3; plasma SCI, n = 3; plasma sham, n = 3).

miRNA expression levels were quantified as CPM. Differentially expressed miRNAs (DEmiRNAs) were identified based on a fold change ≥2 and an FDR-adjusted *P* < 0.05.

### miRNA validation by qPCR

On postoperative day 3, CSF (100 µL each) was collected from sham (n = 4) and SCI (n = 4) rats. EVs were isolated as described above, and total RNA was extracted. cDNA was synthesized using a TaqMan MicroRNA Reverse Transcription Kit (Thermo Fisher Scientific, Waltham, MA, USA). PCR reaction mixtures were prepared in 96-well plates at a total volume of 20 µL/well, consisting of 18.67 µL reaction mix and 1.33 µL of 1:10-diluted cDNA. Each sample was run in quadruplicates on a StepOnePlus Real-Time PCR System (Applied Biosystems, Waltham, MA, USA). miR‑9a‑3p expression was quantified using the TaqMan MicroRNA Assay for hsa‑miR‑9* (Assay ID 002231; stem-loop Accession No. MI0000466), which corresponds to the mature miR‑9‑3p sequence in rat, mouse, and human. cel‑miR‑39 (Assay ID 000200; stem-loop Accession No. MI0000010; Thermo Fisher Scientific) was used as the internal control. Relative expression levels were calculated using the 2^−ΔΔCt^ method.

### qPCR analysis of spinal cord and brain tissues

Following perfusion with saline for approximately 2 min, the spinal cord was extracted, and both the spinal cord and brain were rapidly frozen in liquid nitrogen (brain, n = 4; C1, n = 5; Th9, n = 4; Th10, n = 5; Th11, n = 4). The slight variation in sample numbers across regions was due to technical difficulties encountered during tissue dissection and RNA extraction. RNA extraction, cDNA synthesis, and qPCR analysis were conducted as described in the “miRNA validation by qPCR” section. miR‑9a‑3p expression at the lesion site in the spinal cord and in the brain was quantified using the TaqMan MicroRNA Assay for hsa‑miR‑9 (Assay ID 002231; stem-loop Accession No. MI0000466; Thermo Fisher Scientific), with U6 snRNA (Assay ID 001973; NCBI Accession No. NR_004394; Thermo Fisher Scientific) used as the endogenous control. Relative expression was calculated using the 2^−ΔΔCt^ method.

### FISH and immunohistochemistry

Sham and SCI rats (n = 4 each) were killed on postoperative day 3 to collect the brain and spinal cord tissues. After tissue fixation and sectioning, FISH was performed using an miRNAscope Assay Kit (Cosmo Bio Co., Ltd, Tokyo, Japan). For detection of rno-miR-9a-3p, we used the miRNAscope Target Probe - SR-rno-miR-9a-3p-S1 (catalog no. 1560851-S1, ADC, Newark, CA, USA). As controls, we used the miRNAscope Negative Control Probe - SR-Scramble-S1 (catalog no. 727881-S1, ADC) and the miRNAscope Positive Control Probe - SR-RNU6-S1 (catalog no. 727871-S1, ADC). Immunostaining was performed using primary antibodies against HuC/D (Thermo Fisher Scientific), GFAP (Proteintech, Rosemont, IL, USA), Olig2 (R&D Systems, Minneapolis, MN, USA), and Iba1 (FUJIFILM Wako, Osaka, Japan). For quantification of miR-9a-3p expression, one section per animal was analyzed. The percentage of miR-9a-3p-positive area within DAPI+ and GFAP+ regions was calculated using ImageJ software with identical thresholding parameters applied to all images. Negative control probes (scrambled probes for miR-9a-3p) were stained under the same conditions and used to subtract background signal from the miR-9a-3p channel. Although all slides were processed simultaneously using identical staining protocols, we observed minor inter-slide variability in signal intensity. Therefore, we did not perform direct quantitative comparisons between sham and SCI groups. Instead, quantification was limited to within-group analyses (e.g., cell-type comparisons), which allowed us to assess the relative localization of miR-9a-3p signal in a consistent and internally controlled manner. Detailed staining protocols are provided in the Supplementary Materials.

### Human CSF samples

This study was approved by the Ethics Committee of Keio University (approval no. 20231158) and was conducted in accordance with the relevant ethical guidelines. CSF samples were obtained from participants in a phase I/II clinical trial of KP-100IT for acute SCI (ClinicalTrials.gov identifier, NCT02193334)^35^, after obtaining written informed consent. Additional CSF samples were obtained from the National Center of Neurology and Psychiatry (NCNP) biobank, a member of National Center Biobank Network (NCBN)^74^. Usage of NCNP biobank samples was approved by both the utilization committee (No. NCNPBB-0132) and the ethics committee of the National Center of Neurology and Psychiatry (No. A2012-091). These control samples were residual CSF specimens obtained from individuals who underwent lumbar puncture due to clinical indications but were not diagnosed with any central nervous system (CNS) diseases. According to the center’s internal criteria, inflammatory, autoimmune, infectious, and neoplastic CNS conditions were excluded, minimizing the likelihood of disease-related bias in CSF miRNA expression. For the SCI group, participants were classified into recovery or non-recovery based on neurological assessments using the modified Frankel grading system. Recovery was defined as improvement from grade A to B or C, whereas non-recovery was defined as remaining at grade A. All 16 patients with SCI had traumatic cervical injuries and were classified as modified Frankel grade A at baseline. Most were injured by high-energy falls and underwent posterior spinal fixation. Among them, detailed perioperative data were available for 13 patients, and CSF was collected preoperatively in 12 of these cases. No substantial clinical differences, such as age, neurological level, or injury mechanism, were observed between the recovery and non-recovery groups. Initially, 20 samples (control, n = 4; non-recovery, n = 11; recovery, n = 5) were included in the dataset. However, two samples (PR434208a and PR434213a) were identified as outliers using RPCA (PcaHubert) and were excluded from further analysis. RPCA detected these samples as significant outliers based on their Mahalanobis distances (37.67 and 64.58), which exceeded the threshold derived from the IQR criterion (median + 1.5 × IQR). Thus, the baseline characteristics (Table 1) were obtained, and all downstream analyses, including PCA and differential expression analysis, were performed after excluding outliers. Supplementary Table 4 summarizes the clinical data of all 20 human subjects, including both the excluded and analyzed samples. Ultimately, 18 samples (control, n = 4; non-recovery, n = 9; recovery, n = 5) were analyzed (Supplementary Table 5). From each subject, 400 µL of CSF was collected. EVs and total RNA were extracted as described for animal samples, and miRNA sequencing was performed using Takara Bio. All participants provided written informed consent before sample collection. Participant recruitment and data handling complied with international and institutional ethical guidelines, ensuring equitable inclusion without involvement of vulnerable populations. These procedures fulfill the requirements for ethical inclusion and transparency in biomedical research as outlined in the Nature Portfolio guidelines.

### Introduction of miR-9-3p into Human Motor Neurons

Human spinal motor neurons were differentiated from the human induced pluripotent stem cell (hiPSC) line 201B7 (RRID: CVCL_A324), obtained from the Center for iPS Cell Research and Application (CiRA), Kyoto University. The cells were differentiated into spinal lower motor neurons using a rapid and efficient induction protocol, which combined small molecule pretreatment (SB431542, CHIR99021, and dorsomorphin) with Sendai virus-mediated transduction of transcription factors LHX3, NGN2, and ISL1. This protocol enables spinal motor neuron differentiation with approximately 80% efficiency within two weeks, and has been validated in prior ALS disease modeling studies^75–77^. Day-14 motor neuron derivatives were used for all experiments. Two groups were prepared: a scramble control group and a miR-9-3p overexpression group, each with three replicates (n = 3 per group). The control group corresponded to sample IDs PR434501 to PR434503, and the miR-9-3p overexpression group corresponded to PR434504 to PR434506. Motor neurons were cultured in vitro and transduced with custom-designed recombinant lentiviruses (LV), which were purchased from Vector Builder and used at a multiplicity of infection (MOI) of 5. An LV containing the miR-9-3p sequence (catalog no. LVL [VB240709-1008vye]; lot no. 240724LVX11; titer, 3.13 × 10^9^ TU/mL) was used for the LV–miR-9-3p group; an LV with a scrambled sequence (catalog no. LVC [VB010000-0009mxc]-b; lot no. 240223LVJ79; titer, 1.73 × 10^9^ TU/mL) served as the control. Viruses were suspended in Hanks’ balanced salt solution and stored at −80 °C until use. Following transduction, total RNA was extracted using the miRNeasy Mini Kit (Qiagen, Hilden, Germany). miR-9-3p expression (normalized to U6) was quantified by qPCR using the 2^−ΔΔCt^ method. For transcriptome analysis, RNA samples were outsourced to Takara Bio for sequencing on an Illumina platform.

### Statistics and reproducibility

For miRNA sequencing data, the Qiagen RNA Analysis Pipeline (RAP) was used to identify DEmiRNAs, applying a fold change cutoff ≥2 and an FDR-adjusted *P* < 0.05. For human samples, the *P* values were further adjusted by doubling the FDR *P* values produced using RAP software.

To ensure data quality, the RPCA was used to detect statistical outliers in the human CSF dataset. Outliers were identified based on Mahalanobis distances and an IQR-based threshold (median + 1.5 × IQR) and subsequently excluded from all downstream analyses. A different exclusion criterion was applied to the rat CSF and plasma miRNA sequencing data, in which samples with extremely low miRNA concentrations (<20 pg/µL) were removed to ensure sufficient RNA input for sequencing.

To evaluate potential batch effects, PCA was performed, and PC1 and PC2 scores were compared between sequencing batches (first vs. second) using Welch’s t-tests. Additionally, PERMANOVA was conducted to assess the overall batch effects across the entire PCA space.

For the overexpression model in human-derived motor neurons, raw RNA sequencing data were analyzed using RaNA-Seq (University of Salamanca, Salamanca, Spain) to identify differentially expressed genes, which were then subjected to functional annotation using the DAVID v6.8. The GO terms for biological process, cellular component, and molecular function with a Benjamini–Hochberg-adjusted *P* < 0.05 were deemed significant. GO terms identified using DAVID were visualized in Cytoscape (v3.10.2) using the Enrichment Map plugin (v3.4.0), followed by clustering using the AutoAnnotate plugin (v1.5.1).

GSEA was performed using GSEA software (v4.3.3) and the Molecular Signatures Database (MSigDB) c5.all.v2024.1.Hs.symbols.gmt (GO-based sets) and h.all.v2024.1.Hs.symbols.gmt (Hallmark sets). The Human_Ensembl_Gene_ID_MSigDB.v2024.1.Hs.chip was used as the reference platform. The number of permutations was set at 1000, and the default parameters were applied.

For qPCR validation of miRNA expression, two-sided unpaired Student’s *t* tests were performed (*P* < 0.05). Two-way ANOVA was used for the quantitative analysis of the FISH data, using GraphPad Prism (v10; GraphPad Software, San Diego, CA, USA).

Assistance with language editing and phrasing in the preparation of this manuscript was provided by a large language model (ChatGPT, OpenAI). The model was used solely to improve English clarity and grammar. All scientific content, interpretations, and conclusions were produced, verified, and approved by the authors.

## Supplementary information

Supplementary Information

Description of Additional Supplementary Files

Supplementary Data 1

Supplementary Data 2

Supplementary Data 3

Supplementary Data 4

Supplementary Data 5

Supplementary Data 6

Supplementary Data 7

Supplementary Data 8

Supplementary Data 9

Supplementary Data 10

Reporting Summery

## Data Availability

All data supporting the findings of this study are available in the main text or supplementary materials. Transcriptomic datasets have been deposited in NCBI BioProject database as follows: the human CSF miRNA-seq dataset is available under accession number PRJNA1218608, the rat CSF and plasma miRNA-seq dataset under PRJNA1221015, and the human motor neuron RNA-seq dataset under PRJNA1218619. Additional materials, including the code and reagents, are available from the corresponding author upon reasonable request, provided no immediate access requirements are imposed by journal policy or editorial mandate. Detailed quantitative data used in this study—including qPCR values, FISH-based microscopic measurements, and CPM values from miRNA sequencing—are provided in the Supplementary Data. For the rat dataset, Supplementary Data 1 contains the PCA score matrix derived from cerebrospinal fluid (CSF) extracellular vesicle (EV) miRNA sequencing, while Supplementary Data 2 provides the raw counts per million (CPM) values for individual miRNAs detected in both CSF- and plasma-derived EVs. Differential expression analyses for CSF and plasma miRNAs are consolidated in Supplementary Data 3, which includes two worksheets presenting normalized expression levels, false discovery rate (FDR)-adjusted P-values, and log₂ fold changes: Sheet 1 contains CSF data, and Sheet 2 contains plasma data. Supplementary Data 4 presents quantitative PCR (qPCR) results for miR-9a-3p levels in rat CSF-derived EVs. Supplementary Data 5 reports qPCR-based expression profiles of miR-9a-3p across distinct regions of the central nervous system, including the primary motor cortex, cervical spinal cord (C1), and thoracic spinal cord levels (Th9, Th10 at the lesion site, and Th11 caudal to the lesion); each region is provided in a separate worksheet within the same file (Sheets 1–5). Supplementary Data 6 includes cellular localization data of miR-9a-3p, based on fluorescence in situ hybridization (FISH) and immunostaining in astrocytes, neurons, oligodendrocytes, and microglia from both the spinal cord and brain. For human CSF samples, Supplementary Data 7 presents CPM values from EV miRNA-seq, with two worksheets comparing miRNA expression profiles: Sheet 1 contains data comparing control and non-recovery SCI subjects, while Sheet 2 compares non-recovery and recovery groups. Supplementary Data 8 contains ΔΔCT values from in vitro qPCR analysis validating miR-9-3p overexpression using adeno-associated virus (AAV) vectors. Supplementary Data 9 presents transcriptomic changes in human motor neurons following lentiviral miR-9-3p transduction, shown as CPM values from RNA sequencing. Supplementary Data 10 provides gene ontology (GO) enrichment analysis results based on the motor neuron RNA-seq data. It includes two worksheets: Sheet 1 contains functional categories enriched among highly expressed genes, and Sheet 2 contains those enriched among lowly expressed genes.
