# Supplementary material for: Cerebrospinal fluid extracellular vesicle-derived miR-9-3p in spinal cord injury with neuroprotective implications and biomarker development
Source: Commun Biol. 2025 Oct 27;8:1498. doi: 10.1038/s42003-025-08947-3 (PMC12559747; doi:10.1038/s42003-025-08947-3)
Supplement: Supplementary file 1 — Supplementary Information [file 42003_2025_8947_MOESM1_ESM.pdf]

Supplementary Materials for "Cerebrospinal fluid extracellular vesicle-derived miR-9-3p in spinal cord injury with neuroprotective implications and biomarker development"

Authors: Tomoharu Tanaka[1]†, Satoru Morimoto[2]†, Keitaro Ito[1], Kaori Yasutake[1], Chris Kato[2], Munehisa Shinozaki[2], Kota Suda[3], Takeshi Maeda[4], Yoshiyuki Yato[5], Masaya Nakamura[1], Hideyuki Okano[2]*, and Narihito Nagoshi[1]*

SUPPLEMENTARY METHODS

Fluorescent In Situ Hybridization (FISH) and immunohistochemistry

Four rats from each group (sham and SCI groups) were used for analyses. On postoperative day 3, the animals were perfused with saline, followed by fixation with 4% paraformaldehyde (PFA) phosphate buffer solution. The brain and spinal cord tissues were harvested, fixed in 4% PFA at 4°C for 24 h, and then cryoprotected by sequential immersion in 10%, 20%, and 30% sucrose at 4°C (18 h each). After fixation, further fixation was performed in dry ice acetone, and the tissues were embedded in Optimal Cutting Temperature compound. Tissue sections (12 μm thick) were prepared using a cryostat and stored at −80°C until use.

FISH was performed using the miRNAscope Assay Kit (Cosmo Bio). The frozen sections

were washed in phosphate buffered saline (PBS) for 5 min, dried at 60°C for 1 h, and fixed in 4% PFA at 4°C for 15 min. Sections were sequentially dehydrated in 50%, 75%, and 100% ethanol (with two changes in 100% ethanol). Next, the sections were treated with 10% neutral buffered formalin (NBF) at room temperature for 18 h, rinsed in distilled water for 2 min, and dried at 60°C for 5 min. RNAscope Peroxidase (Cosmo Bio) was applied for 10 min at room temperature for clearing, followed by RNAscope Target Retrieval (Cosmo Bio) at 98°C–102°C for 5 min to enable antigen retrieval. After cooling, sections were incubated with RNAscope Protease Plus (Cosmo Bio) at room temperature for 20 min. Hybridization with miRNAscope probes (Cosmo Bio) was performed in a humidified chamber at 40°C for 2 h. Subsequent amplification steps (AMP 1–6) and chromogenic detection were performed according to the manufacturer's protocol.

Following FISH, the sections were blocked with 4% skim milk in PBS at room temperature for 1 h and incubated with the following primary antibodies: HuC/D (Invitrogen, catalog no. A21271, 1:100), GFAP (Proteintech, catalog no. 60190-1-LG, 1:1000), Olig2 (R&D Systems, catalog no. AF2418, 1:150), and Iba1 (Wako, catalog no. 019-19741, 1:250). After washing three times in PBS (5 min each), the following secondary antibodies were applied: goat anti-mouse IgG Alexa Fluor 488 (Invitrogen, catalog no. A11029, 1:1000) for HuC/D, goat anti-

mouse IgG Alexa Fluor 488 (Invitrogen, catalog no. A11029, 1:1000) for GFAP, donkey anti-goat IgG Alexa Fluor 488 (Invitrogen, catalog no. A11055, 1:1000) for Olig2, and goat anti-rabbit IgG Alexa Fluor 488 (Invitrogen, catalog no. A11034, 1:1000) for Iba1. The sections were washed thrice in PBS (5 min each). Finally, the signals were detected and visualized according to the manufacturer's standard protocol, and images were obtained using a confocal laser microscope (Leica TCS SP8; Leica, Wetzlar, Germany).

SUPPLEMENTARY FIGURES

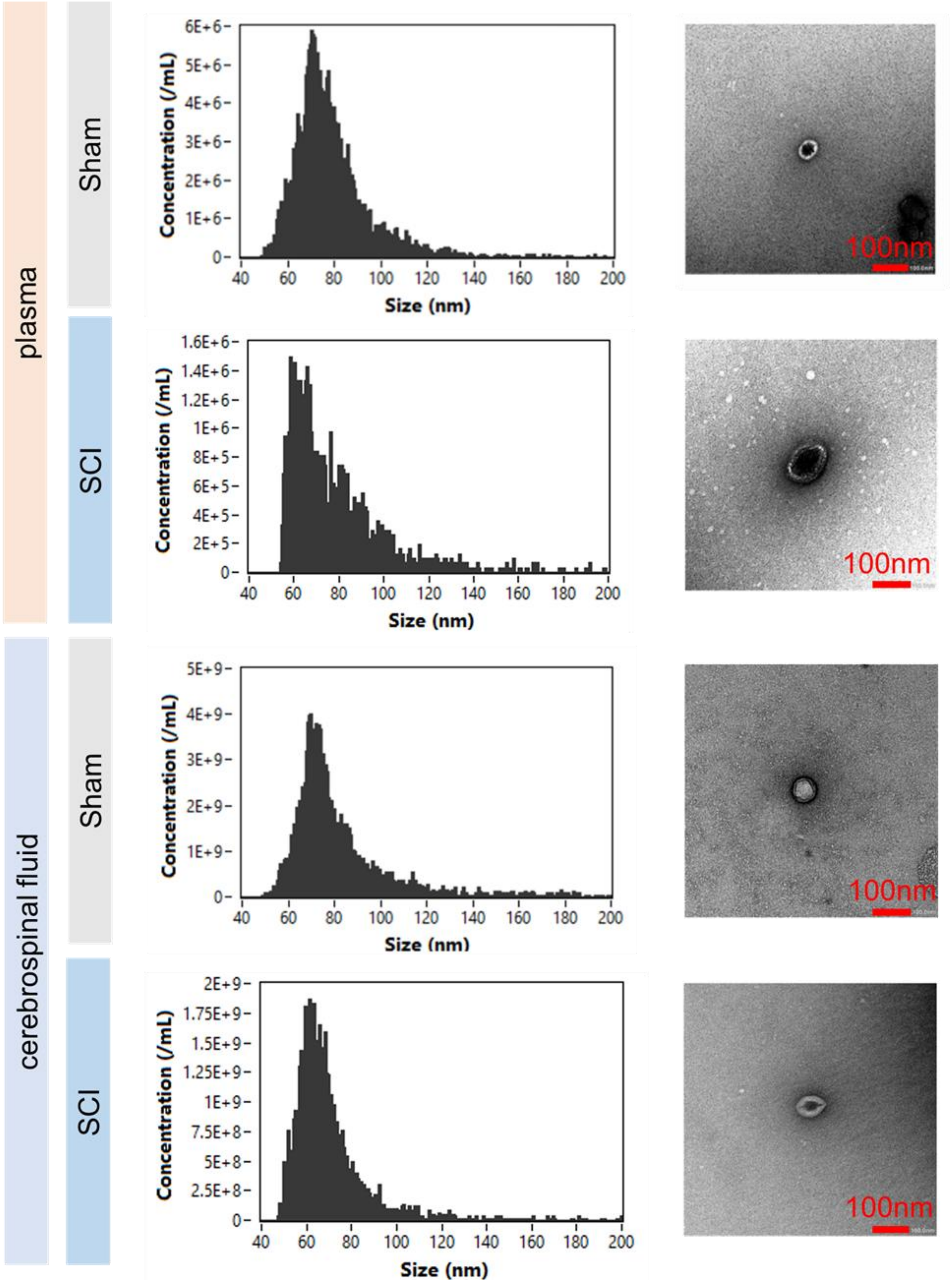
plasma
Sham
SCI
cerebrospinal fluid
Sham
SCI
Concentration (/mL)
Size (nm)
100nm

Supplementary Figure 1. Characterization of EVs derived from CSF and plasma by NanoFCM and TEM. The particle sizes ranged from 50 to 200 nm, with a peak at 70–80 nm (NanoFCM). Electron microscopy confirmed the presence of lipid bilayer vesicles.

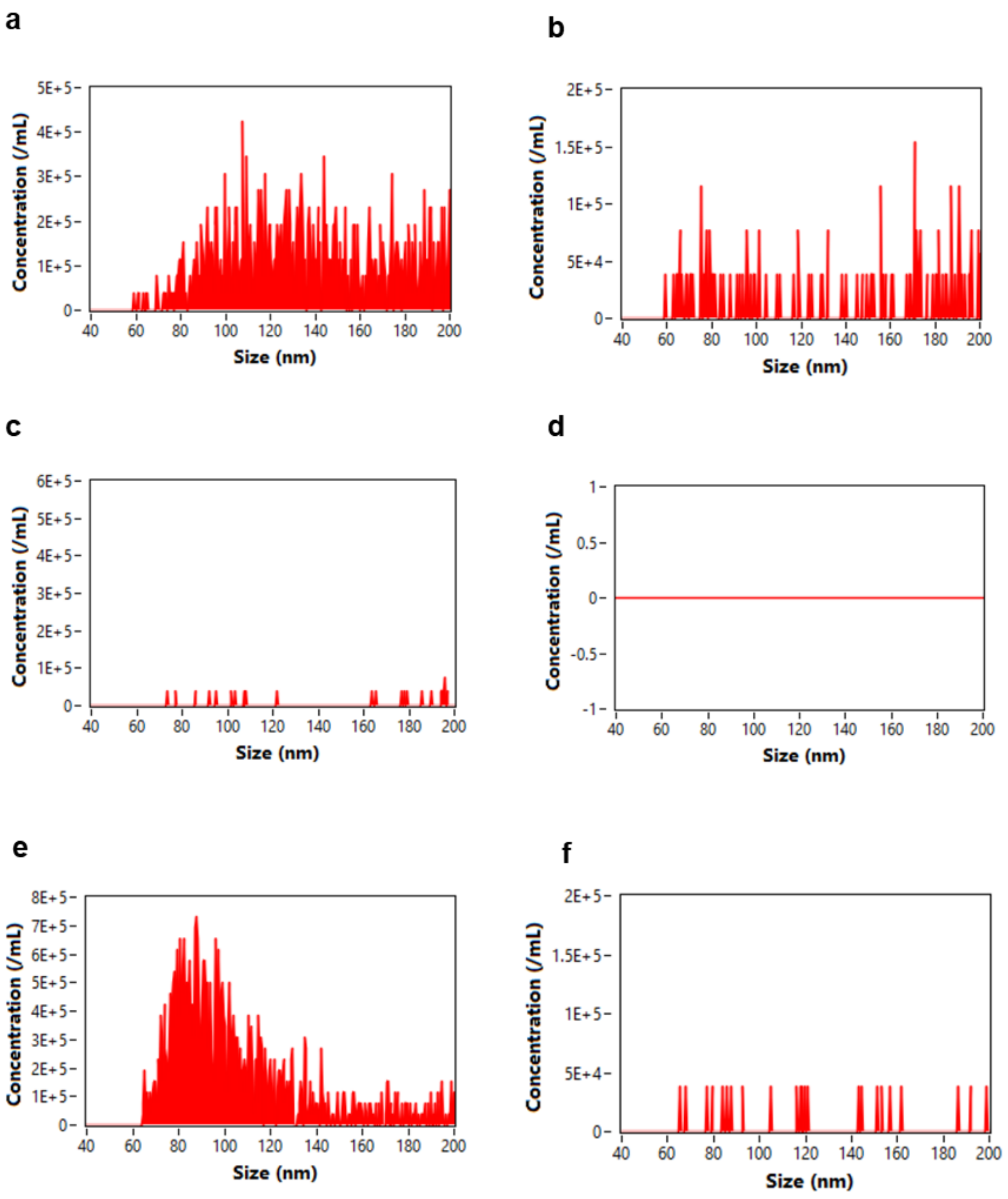

Supplementary Figure 2. Validation of tetraspanin-positive extracellular vesicles by NanoFCM. Particle size distribution profiles of fluorescently labeled extracellular vesicles were analyzed using NanoFCM. The y-axis indicates particle concentration (particles/mL),

and the x-axis indicates particle size (nm). a) CD81 in the stained sample; b) CD81 in the PBS control; c) ALIX in the stained sample; d) ALIX in the PBS control; e) TSG101 in the stained sample; f) TSG101 in the PBS control. Fluorescently labeled particles were detected predominantly in the 50–200 nm range in all stained samples.

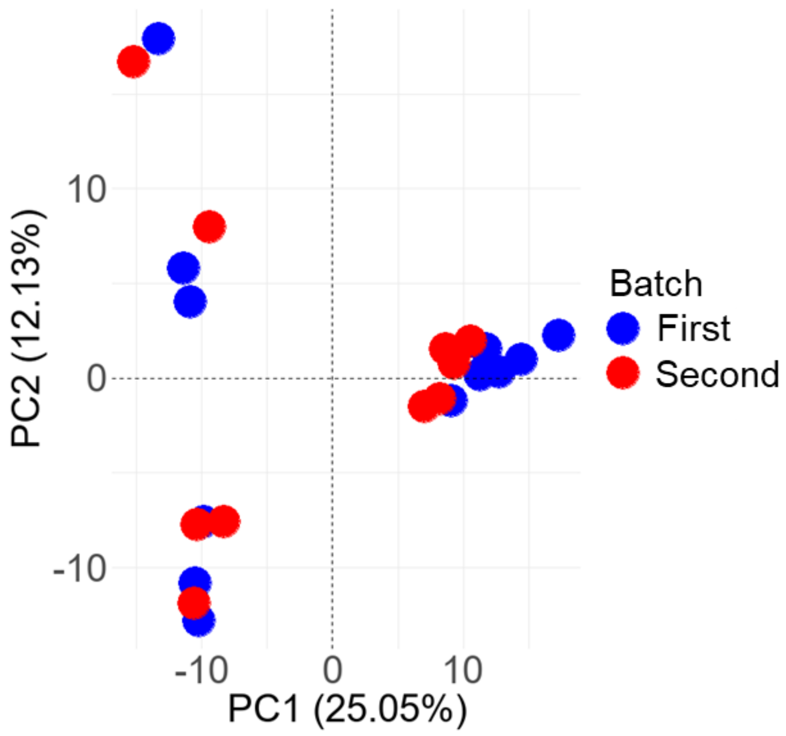

Supplementary Figure 3. PCA for batch effect evaluation. PCA of miRNA sequencing data with samples colored according to sequencing batch (first vs. second). No distinct clustering was observed between batches, indicating minimal batch effects. Statistical analysis further confirmed that there were no significant differences between batches (PC1: $P = 0.704$, PC2: $P = 0.970$; PERMANOVA $R^2 = 0.0048$, $P = 0.879$). Therefore, all samples were analyzed without batch correction.

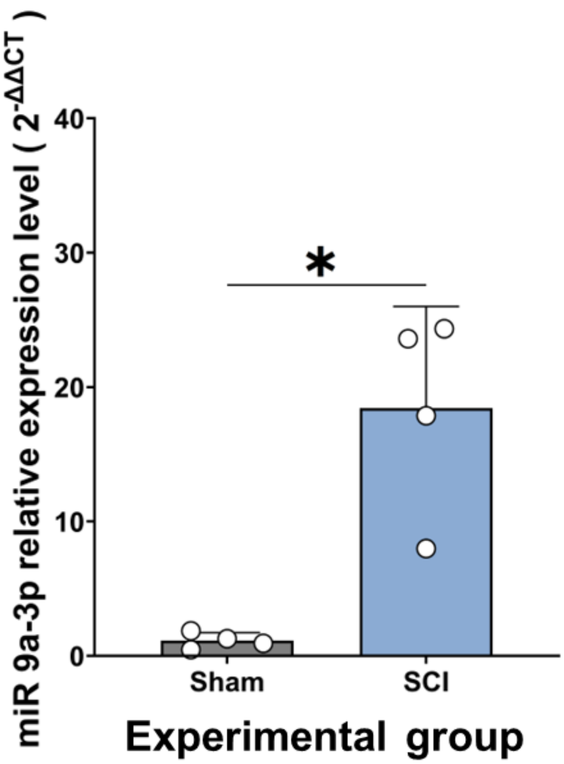

Supplementary Figure 4. Validation of miRNA sequencing results by real-time reverse transcription-polymerase chain reaction. miR-9a-3p expression in CSF-derived EVs was significantly higher in the SCI group than in the sham group (sham vs. SCI, $P = 0.0038$, two-sided unpaired Student's t-test), confirming the reproducibility of the miRNA-seq findings. Data are shown as the mean ± standard error (n = 4). Error bars represent mean ± SD.

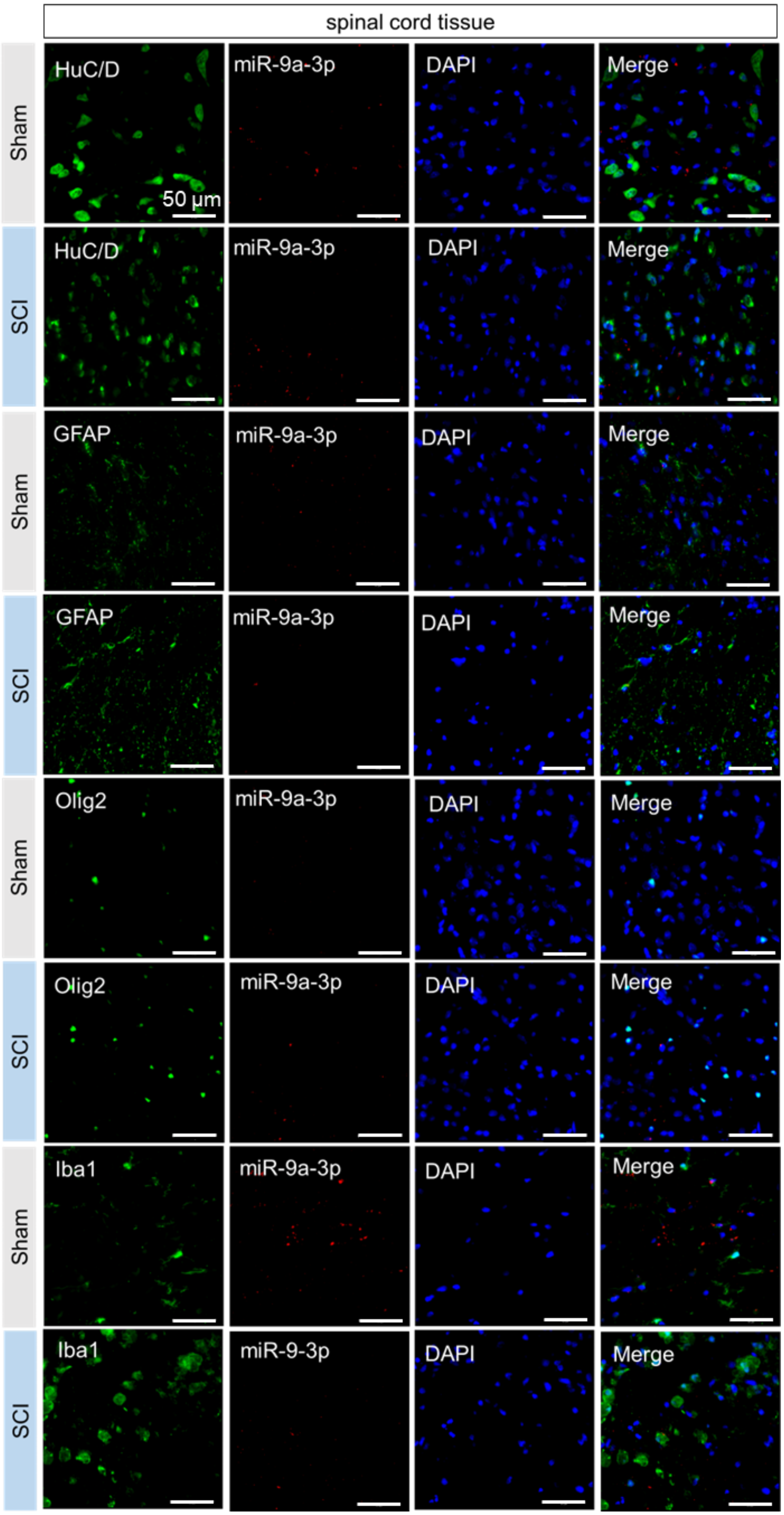

spinal cord tissue
Sham
HuC/D
50 μm
miR-9a-3p
DAPI
Merge
SCI
HuC/D
miR-9a-3p
DAPI
Merge
Sham
GFAP
miR-9a-3p
DAPI
Merge
SCI
GFAP
miR-9a-3p
DAPI
Merge
Sham
Olig2
miR-9a-3p
DAPI
Merge
SCI
Olig2
miR-9a-3p
DAPI
Merge
Sham
Iba1
miR-9a-3p
DAPI
Merge
SCI
Iba1
miR-9-3p
DAPI
Merge

Supplementary Figure 5. FISH and immunostaining of miR-9a-3p in the spinal cord. FISH and immunostaining were performed to visualize the miR-9a-3p expression in neurons, astrocytes, oligodendrocytes, and microglia within the spinal cord. miR-9a-3p signals were predominantly co-localized with astrocytes in both cytoplasm and nucleus.

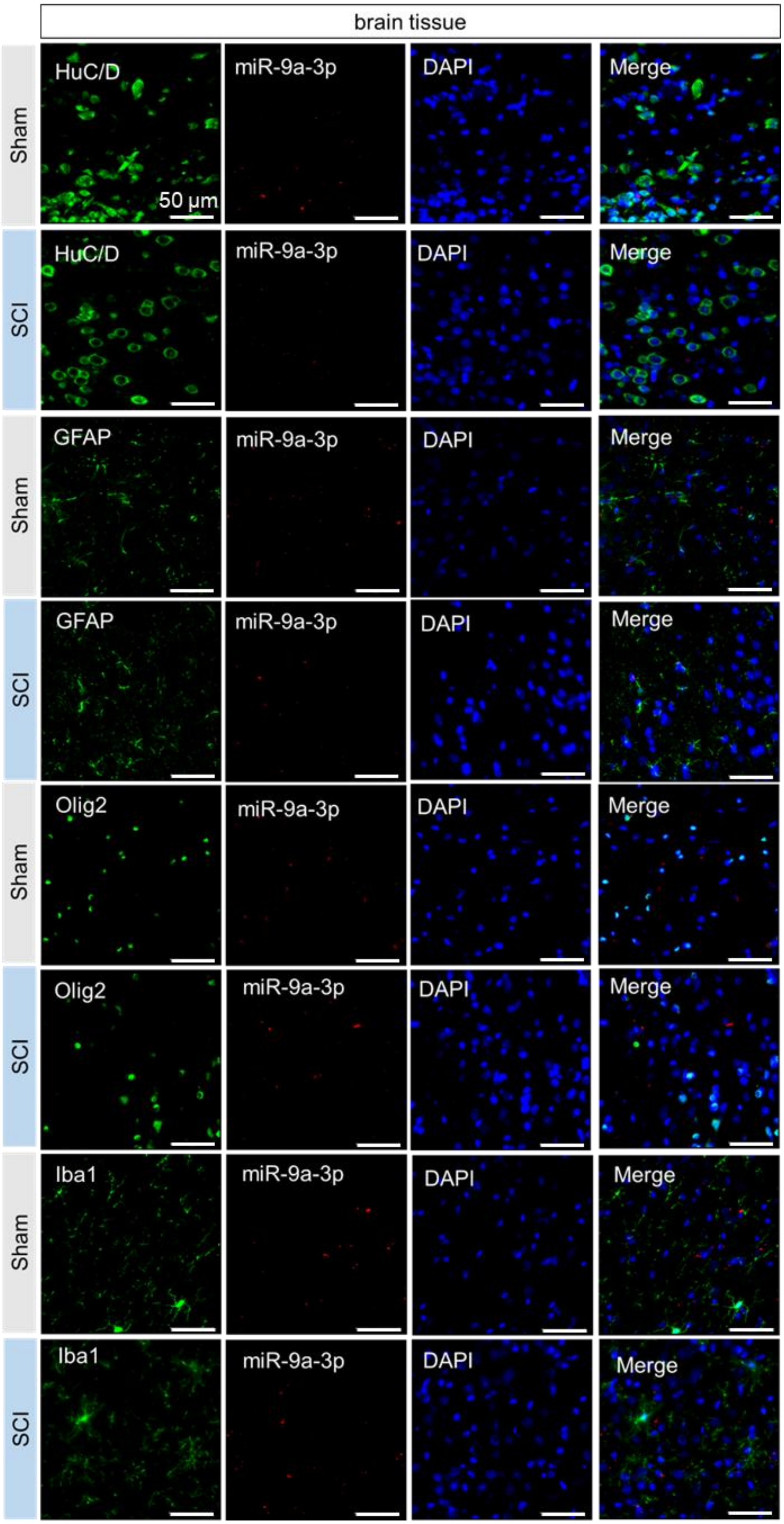
brain tissue
Sham
SCI
HuC/D
GFAP
Olig2
Iba1
miR-9a-3p
DAPI
Merge
50 μm

Supplementary Figure 6. FISH and immunostaining of miR-9a-3p in the brain. FISH and immunostaining were performed to visualize miR-9a-3p expression in neurons, astrocytes, oligodendrocytes, and microglia within the brain. miR-9a-3p signals were predominantly co-localized with astrocytes in both cytoplasm and nucleus.

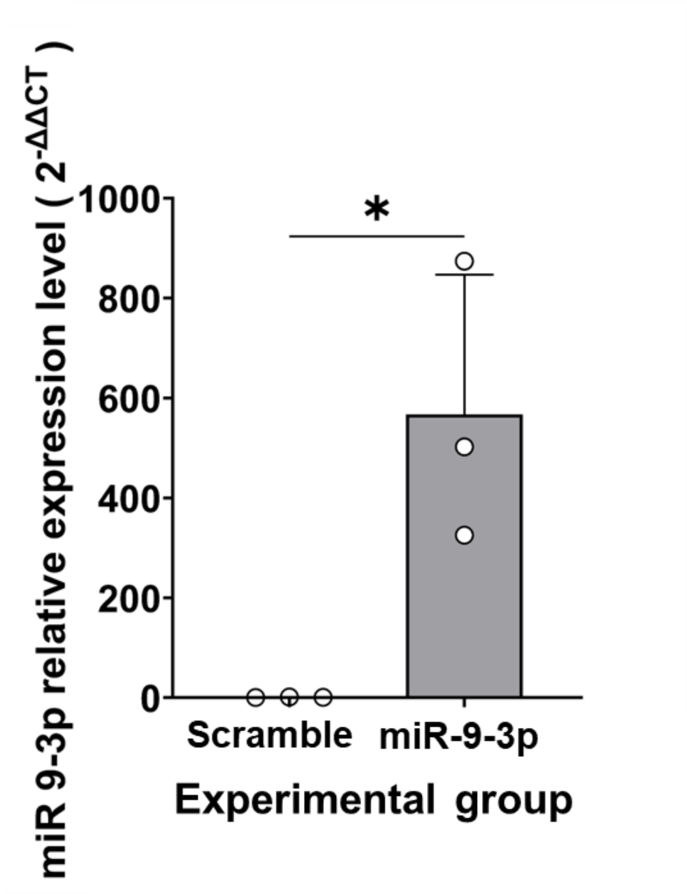

Supplementary Figure 7. qPCR analysis of miR-9-3p expression in the scramble and miR-9-3p groups. qPCR was performed to assess miR-9-3p expression in the scrambled (n = 3) and miR-9-3p (n = 3) groups. The miR-9-3p levels were significantly higher in the miR-9-3p group (P = 0.0248). Error bars represent mean ± SD.

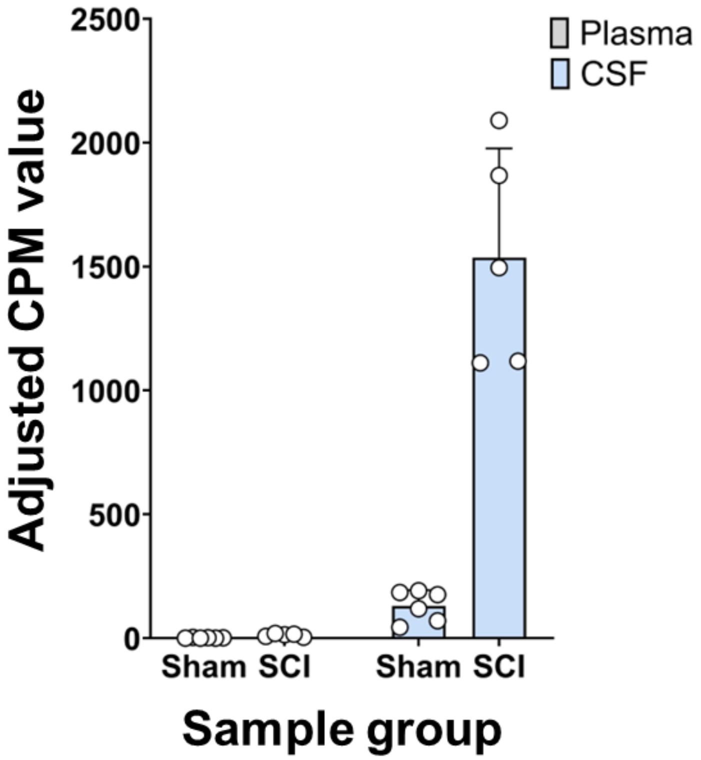

Supplementary Figure 8. Quantification of miR-9a-3p expression levels in CSF- and plasma-derived EVs based on miRNA sequencing. miRNA sequencing was performed on CSF- and plasma-derived EVs from sham (n = 6) and SCI (n = 5) rats. miR-9-3p expression was markedly higher in the CSF than in the plasma, suggesting its predominant localization in the CNS. Error bars represent mean ± SD.

SUPPLEMENTARY TABLES

Supplementary Table 1. Red blood cell (RBC) counts in CSF on day 3 post-injury

Sample ID	Sample type	RBC count (10^4/μL)
PR371201a	CSF	<1
PR371202a	CSF	<1
PR371203a	CSF	<1
PR399301a	CSF	<1
PR399302a	CSF	<1
PR399303a	CSF	<1
PR371204a	CSF	<1
PR371205a	CSF	<1
PR371206a	CSF	<1
PR399304a	CSF	<1
PR399305a	CSF	<1

RBC counts were consistently below 1×10^4/μL in all CSF samples from both sham (n = 6) and SCI (n = 5) groups, indicating minimal blood contamination across the samples.

Supplementary Table 2. Quantification of fluorescently labeled EVs from sham CSF by NanoFCM.

		CD81	ALIX	TSG101
Sample	All events	917	62	1245
	Median (nm)	139.2	204.8	97.2
	Mean (nm)	144.2	183.6	109
	Sample concentration (particles/mL)	3.53E+07	2.39E+06	4.80E+07
Control	All events	158	0	44
	Median	170.8	0.2	191.8
	Mean	150.8	NA	162.1
	Sample concentration (particles/mL)	6.09E+06	0.00E+00	1.70E+06

Summary of NanoFCM analysis of fluorescently labeled extracellular vesicles (EVs) isolated from cerebrospinal fluid (CSF) of sham-operated rats. EVs were stained with antibodies against CD81, ALIX, and TSG101. The total number of detected events, median and mean particle sizes (nm), and calculated particle concentrations (particles/mL) are shown for both the stained samples and the PBS controls. EVs positive for all three markers were detected in the sham sample, while minimal or no signal was observed in the PBS control, supporting

the specificity of the labeling and the presence of EVs in the sham CSF.

Supplementary Table 3. miRNA concentration and exclusion status in rat CSF and plasma samples

Sample ID	Sample type	Status	miRNA concentration (pg/μL)	Exclusion status
PR371201a	CSF	Sham	211.8	Yes
PR371202a	CSF	Sham	71.1	Yes
PR371203a	CSF	Sham	198.1	Yes
PR399301a	CSF	Sham	143.4	Yes
PR399302a	CSF	Sham	192.6	Yes
PR399303a	CSF	Sham	95.1	Yes
PR371204a	CSF	SCI	231.2	Yes
PR371205a	CSF	SCI	203.6	Yes
PR371206a	CSF	SCI	246.7	Yes
PR399304a	CSF	SCI	252.5	Yes
PR399305a	CSF	SCI	5.8	No
PR399306a	CSF	SCI	164.7	Yes

PR371207a	Plasma	Sham	252.5	Yes
PR371208a	Plasma	Sham	244.6	Yes
PR371209a	Plasma	Sham	213.9	Yes
PR399307a	Plasma	Sham	54.4	Yes
PR399308a	Plasma	Sham	71.1	Yes
PR399309a	Plasma	Sham	73.9	Yes
PR371210a	Plasma	SCI	183.6	Yes
PR371211a	Plasma	SCI	79.6	Yes
PR371212a	Plasma	SCI	274.2	Yes
PR399310a	Plasma	SCI	205.2	Yes
PR399311a	Plasma	SCI	100.0	Yes
PR399312a	Plasma	SCI	13.9	No

This table summarizes the miRNA concentrations (pg/μL) measured using an Agilent 2100 BioAnalyzer in rat CSF and plasma samples and the exclusion status of each sample. Samples with extremely low miRNA concentrations (<20 pg/μL) were excluded from further analysis.

Supplementary Table 4. Demographic and clinical characteristics of 20 human participants

Sample ID	Age (years)	Sex	Neurological injury level	Injury cause	Situation	Surgery timing relative to CSF	Surgical procedure	Modified Frankel grade at baseline	Modified Frankel grade at follow-up
PR434201a	58	F	No injury	N/A	N/A	N/A	N/A	N/A	N/A
PR434202a	54	F	No injury	N/A	N/A	N/A	N/A	N/A	N/A
PR434203a	53	M	No injury	N/A	N/A	N/A	N/A	N/A	N/A
PR434204	50	M	No injury	N/A	N/A	N/A	N/A	N/A	N/A

a									
PR434205a	69	M	C6-7	Fall	Fell from 1-meter height while pruning in the garden	Before	Reduction and wiring	A	A
PR434206a	70	M	C4-5	Fall	Fell while standing on a table to replace a light bulb at home	Before	Reduction and posterior fixation (Roger's wiring)	A	A
PR434207a	70	F	C7	Fall	Fell down the stairs at home	Before	Reduction and posterior fixation	A	A

PR434208a	52	M	C3,4	Accident	Collision during boat race	N/A	N/A	A	A
PR434209a	60	M	C4-5	Fall	Fell from a 3-meter breakwater while fishing, hitting the head on concrete	Before	Posterior fixation	A	A
PR434210a	49	M	C6-7	Fall	Fell from a 4-meter loft at home	Before	Posterior fixation	A	A
PR434211a	67	M	C5	Fall	Fell down stairs while trying to change a car tire	Before	Posterior fixation	A	A
PR434212	70	F	C5	Fall	Fell down stairs inside	Before	Posterior	A	A

a					the house		fixation		
PR434213 a	61	M	C6,7	Fall	After drinking, stumbled while getting out of a taxi and fell backwards	Before	Posterior fixation	A	A
PR434214 a	24	M	C7	Fall	Failed landing during trampoline performance at circus (work-related accident)	After	Anterior and posterior fixation	A	A
PR434215 a	20	M	C5	Accid ent	Thrown by an opponent during	Before	Posterior fixation and	A	A

					wrestling practice		spinal canal decompression		
PR434216a	68	F	C4-6	Fall	Fell about 2 meters when an elevator was not present during entry	N/A	N/A	A	B2
PR434217a	60	M	C3-4	Accident	Trapped by a falling tree while cutting	N/A	N/A	A	B1
PR434218a	62	M	C6-7	Fall	Slipped off a stone wall while washing a car and fell 2 meters onto asphalt	Before	Posterior fixation	A	B2

PR434219a	53	M	C6-7	Fall	Fell from 3–4 meter height while pruning trees (work-related accident)	Before	Posterior fixation	A	B2
PR434220a	72	M	C4-5	Fall	Fell backwards from a 1-meter stepladder	Before	Posterior fixation and spinal canal decompression	A	C1

This table summarizes the participants' demographics and clinical outcomes, including age, sex (F, female; M, male), neurological injury level, and modified Frankel grade at baseline and follow-up. Control participants with no injuries are marked "N/A" (not applicable) for modified Frankel grades, whereas injured participants are classified by neurological level and modified Frankel grades, allowing for a clear distinction in clinical characteristics.

Supplementary Table 5. RPCA results for outlier detection

Sample ID	Robust Mahalanobis distance	*P*-value	Included in final analysis
PR434201a	2.35	0.218	Yes
PR434202a	1.98	0.267	Yes
PR434203a	3.12	0.154	Yes
PR434204a	2.84	0.178	Yes
PR434205a	5.43	0.067	Yes
PR434206a	4.79	0.089	Yes
PR434207a	3.91	0.129	Yes
PR434208a	37.67	3.21×10^{-7}	No
PR434209a	5.02	0.074	Yes
PR434210a	3.76	0.135	Yes
PR434211a	4.28	0.107	Yes
PR434212a	3.47	0.148	Yes
PR434213a	64.58	5.84×10^{-12}	No

PR434214a	2.91	0.172	Yes
PR434215a	3.41	0.151	Yes
PR434216a	4.55	0.095	Yes
PR434217a	5.14	0.071	Yes
PR434218a	4.92	0.078	Yes
PR434219a	3.88	0.131	Yes
PR434220a	4.67	0.092	Yes

This table summarizes the results of the RPCA-based outlier detection. The robust Mahalanobis distance and corresponding *P*-value were calculated for each sample. Samples with extreme distances exceeding the predefined threshold (median + 1.5 × IQR) were considered statistical outliers and excluded from downstream analyses. Two samples, PR434208a and PR434213a, were identified as significant outliers, with robust distances of 37.67 and 64.58, and *P*-values of 3.21×10^{-7} and 5.84×10^{-12}, respectively. All the other samples were retained for further analysis.

Supplementary Table 6. Previously reported biomarkers reflecting the degree of natural recovery in human SCI

Biomarker	Sampling	Patient groups and sample sizes	Sampling time (post-injury)	Endpoint of functional assessment	AUC	Sensitivity	Specificity	Reference
miR9-3p in EVs	CSF	No recovery (modified Frankel A→A, n=9)/recovery (A→B/C, n=5)	72 hours	2 years	0.8	80	89	This study
NFL	Serum	No recovery (AIS A→A, n=49)/recovery (A→B/C/D, n=25)	72 hours	6 months	0.83	76	77	1
GFAP	Serum		72 hours	6 months	0.8	72	77	
NFL	CSF		72 hours	6 months	0.89	81	79	
GFAP	CSF		72 hours	6 months	0.87	78	80	

Machine learning–based proteomics analysis	CSF	AIS recovery group (n=51)/non-recovery group (n=60)	48 hours	6 months	0.91	90	85	2
Cluster 1: CD14−/CD16+/IL10+/CXCR4int monocytes	peripheral blood	AIS recovery group (n=9)/non-recovery group (n=9)	24 hours	3 months	0.726	N/A	N/A	3
A subset of 30 CSF miRNAs,	CSF	No recovery (AIS A→A, n=12)/recovery (A→B/C, n=12)	24 hours	6 months	0.75	N/A	N/A	4

inflammatory index (IL-6, IL-8, MCP-1)	CSF	No recovery (AIS A→A, n=22)/recovery (A→B/C, n=7)	24 hours	6 months	0.883	72.7	92.3	5
Structural Index (Tau, S100b, GFAP)	CSF		24 hours	6 months	0.857	68.2	92.3	
IL-6	CSF		24 hours	6 months	N/A	N/A	N/A	
IL-8	CSF		24 hours	6 months	N/A	N/A	N/A	
MCP-1	CSF		24 hours	6 months	N/A	N/A	N/A	
Tau	CSF		24 hours	6 months	N/A	N/A	N/A	
S100β	CSF		24 hours	6 months	N/A	N/A	N/A	
GFAP	CSF		24 hours	6 months	N/A	N/A	N/A	

Combination of IL-6 and S100β	CSF	Predicts patients with limited motor score recovery in AIS A	24 hours	6 months	N/A	N/A	N/A	
NFL	Serum	Good recovery (n=14, motor score ≥median)/poor recovery (n=13, motor score <median) groups	7-Day Serum NFL Cumulative Load	12 months	0.83	N/A	N/A	6
TNF-α	Serum	AIS recovery group (n=7)/AIS non-recovery group (n=16)	9 hours	3 months	N/A	N/A	N/A	7

Biochemical prediction model (S100β, GFAP, IL-8)	CSF	No recovery (AIS A→A, n=13)/recovery (A→B/C, n=14)	24 hours	6 months	0.867	N/A	N/A	8
GFAP	CSF	No recovery (AIS A→A, n=5)/recovery (A→B/C, n=2)	24 hours	6 months	N/A	N/A	N/A	9
Tau	CSF		24 hours	6 months	N/A	N/A	N/A	

Summary of biomarkers reported for predicting natural recovery after SCI in human samples. Notably, this study is the first to identify EV-associated miR-9a-3p, which predicts 2-year outcomes with high accuracy (AUC, 0.8; sensitivity, 80%; specificity, 89%), suggesting its potential utility alongside existing biomarkers. N/A indicates unavailable data.

SUPPLEMENTARY REFERENCES

1. Stukas, S. et al. Association of CSF and serum neurofilament light and glial fibrillary acidic protein, injury severity, and outcome in spinal cord injury. Neurology 100, e1221–e1233 (2023).

2. M. A. Skinnider, J. *et al.* Proteomic portraits reveal evolutionarily conserved and divergent responses to spinal cord injury. *Mol. Cell. Proteomics* **20**, 100096 (2021).

3. R. A. Heller, J. *et al.* Predicting neurological recovery after traumatic spinal cord injury by time-resolved analysis of monocyte subsets. *Brain* **144**, 3159–3174 (2021).

4. Tigchelaar, S. et al. MicroRNA biomarkers in cerebrospinal fluid and serum reflect injury severity in human acute traumatic spinal cord injury. J. Neurotrauma 36, 2358–2371 (2019).

5. B. K. Kwon *et al.* Cerebrospinal fluid biomarkers to stratify injury severity and predict outcome in human traumatic spinal cord injury. *J. Neurotrauma* **34**, 567–580 (2017).

6. J. Kuhle *et al.* Serum neurofilament light chain is a biomarker of human spinal cord injury severity and outcome. *J. Neurol. Neurosurg. Psychiatry* **86**, 273–279 (2015).

7. B. Biglari *et al.* A pilot study on temporal changes in IL-1β and TNF-α serum levels after spinal cord injury: the serum level of TNF-α in acute SCI patients as a possible marker for neurological remission. *Spinal Cord* **53**, 510–514 (2015).

8. B. K. Kwon *et al.* Cerebrospinal fluid inflammatory cytokines and biomarkers of injury severity in acute human spinal cord injury. *J. Neurotrauma* **27**, 669–682 (2010).

9. M. H. Pouw *et al.* Structural biomarkers in the cerebrospinal fluid within 24 h after a traumatic spinal cord injury: a descriptive analysis of 16 subjects. *Spinal Cord* **52**, 428–433 (2014).
