# Supplementary material for: Cerebrospinal fluid extracellular vesicle-derived miR-9-3p in spinal cord injury with neuroprotective implications and biomarker development
Source: Commun Biol. 2025 Oct 27;8:1498. doi: 10.1038/s42003-025-08947-3 (PMC12559747; doi:10.1038/s42003-025-08947-3)
Supplement: Supplementary file 2 — Description of Additional Supplementary Files [file 42003_2025_8947_MOESM2_ESM.pdf]

Description of Additional Supplementary Files

File name: Supplementary Data 1
Description: PCA score matrix derived from rat CSF EV miRNA sequencing.

File name: Supplementary Data 2
Description: Raw counts per million (CPM) values for miRNAs detected in CSF- and plasma-derived EVs.

File name: Supplementary Data 3
Description: Differential expression analyses for CSF and plasma miRNAs (normalized expression levels, FDR-adjusted P-values, log2 fold changes).

File name: Supplementary Data 4
Description: qPCR results for miR-9a-3p levels in rat CSF-derived EVs.

File name: Supplementary Data 5
Description: qPCR-based expression profiles of miR-9a-3p across distinct CNS regions (Sheets 1–5).

File name: Supplementary Data 6
Description: Cellular localization data of miR-9a-3p based on FISH and immunostaining in astrocytes, neurons, oligodendrocytes, and microglia.

File name: Supplementary Data 7
Description: CPM values from EV miRNA-seq of human CSF samples comparing control, non-recovery, and recovery groups (two worksheets).

File name: Supplementary Data 8
Description: ΔΔCT values from in vitro qPCR analysis validating miR-9-3p overexpression using AAV vectors.

File name: Supplementary Data 9
Description: Transcriptomic changes in human motor neurons following lentiviral miR-9-3p transduction (CPM values from RNA sequencing).

File name: Supplementary Data 10
Description: Gene ontology (GO) enrichment analysis results based on the motor neuron RNA-seq data (two worksheets: highly vs. lowly expressed genes).
