# Supplementary material for: Cerebrospinal fluid extracellular vesicle-derived miR-9-3p in spinal cord injury with neuroprotective implications and biomarker development
Source: Commun Biol. 2025 Oct 27;8:1498. doi: 10.1038/s42003-025-08947-3 (PMC12559747; doi:10.1038/s42003-025-08947-3)
Supplement: Supplementary file 13 — Reporting Summery [file 42003_2025_8947_MOESM13_ESM.pdf]

nature portfolio

Corresponding author(s): Hideyuki Okano and Narihito Nagoshi, respectively affiliated with the Keio University Regenerative Medicine Research Center and the Keio University School of Medicine

Last updated by author(s): Sep 14, 2025

Reporting Summary

Nature Portfolio wishes to improve the reproducibility of the work that we publish. This form provides structure for consistency and transparency in reporting. For further information on Nature Portfolio policies, see our Editorial Policies and the Editorial Policy Checklist.

Statistics

For all statistical analyses, confirm that the following items are present in the figure legend, table legend, main text, or Methods section.

n/a	Confirmed	
☐	☒	The exact sample size (*n*) for each experimental group/condition, given as a discrete number and unit of measurement
☐	☒	A statement on whether measurements were taken from distinct samples or whether the same sample was measured repeatedly
☐	☒	The statistical test(s) used AND whether they are one- or two-sided<br>*Only common tests should be described solely by name; describe more complex techniques in the Methods section.*
☐	☒	A description of all covariates tested
☐	☒	A description of any assumptions or corrections, such as tests of normality and adjustment for multiple comparisons
☐	☒	A full description of the statistical parameters including central tendency (e.g. means) or other basic estimates (e.g. regression coefficient) AND variation (e.g. standard deviation) or associated estimates of uncertainty (e.g. confidence intervals)
☐	☒	For null hypothesis testing, the test statistic (e.g. *F*, *t*, *r*) with confidence intervals, effect sizes, degrees of freedom and *P* value noted<br>*Give P values as exact values whenever suitable.*
☒	☐	For Bayesian analysis, information on the choice of priors and Markov chain Monte Carlo settings
☒	☐	For hierarchical and complex designs, identification of the appropriate level for tests and full reporting of outcomes
☐	☒	Estimates of effect sizes (e.g. Cohen's *d*, Pearson's *r*), indicating how they were calculated

Our web collection on statistics for biologists contains articles on many of the points above.

Software and code

Policy information about availability of computer code

Data collection	Data collection was performed using the following software: NanoFCM software for nanoparticle characterization, JEOL TEM software for transmission electron microscopy, and StepOne Software v2.3 (Applied Biosystems) for real-time PCR. Fluorescence microscopy images were acquired using Leica THUNDER Imaging System software. Image analysis was conducted using ImageJ.
Data analysis	Data analysis was performed using Qiagen RNA Analysis Pipeline (RAP) for miRNA-seq, RaNA-seq for RNA-seq differential expression, DAVID v6.8 and Cytoscape v3.10.2 with EnrichmentMap and AutoAnnotate plugins for GO analysis, and GSEA v4.3.3 with MSigDB v2024.1 for gene set enrichment analysis. Statistical tests were conducted using GraphPad Prism v10 and R v4.3.1 (including PERMANOVA via the vegan package). Figures were generated using Prism and R.

For manuscripts utilizing custom algorithms or software that are central to the research but not yet described in published literature, software must be made available to editors and reviewers. We strongly encourage code deposition in a community repository (e.g. GitHub). See the Nature Portfolio guidelines for submitting code & software for further information.

Data

Policy information about availability of data

All manuscripts must include a data availability statement. This statement should provide the following information, where applicable:

- Accession codes, unique identifiers, or web links for publicly available datasets
- A description of any restrictions on data availability
- For clinical datasets or third party data, please ensure that the statement adheres to our policy

The sequencing data generated in this study have been deposited in the NCBI Sequence Read Archive (SRA) under the following BioProject accession numbers: PRJNA1218608 (miRNA sequencing of cerebrospinal fluid samples from human spinal cord injury patients), PRJNA1221015 (miRNA sequencing of cerebrospinal fluid and plasma samples from spinal cord injury model rats), and PRJNA1218619 (RNA sequencing of human motor neurons overexpressing miR-9-3p). All datasets will be made publicly available prior to publication.

Research involving human participants, their data, or biological material

Policy information about studies with human participants or human data. See also policy information about sex, gender (identity/presentation), and sexual orientation and race, ethnicity and racism.

Reporting on sex and gender	The sex of all human participants was recorded and is summarized in Table 1. No significant differences in sex distribution were observed between the control, recovery, and non-recovery groups (Fisher's exact test, P = 0.255). Sex was not used as a biological variable in the primary analyses.
Reporting on race, ethnicity, or other socially relevant groupings	All human participants were of Japanese ethnicity. No further analyses by race, ethnicity, or other socially relevant groupings were performed, as all participants belonged to a single ethnic group.
Population characteristics	Study participants included individuals with acute spinal cord injury (SCI) and control donors without neurological injury. All SCI patients were classified as AIS grade A at 72 hours post-injury and were later grouped based on spontaneous recovery status at day 168. The age and sex distribution of participants is presented in Table 1, with no significant differences between groups.
Recruitment	CSF samples from SCI patients were obtained from participants enrolled in a previously conducted clinical trial (NCT02193334) after obtaining written informed consent. Control CSF samples were obtained from healthy donors through the National Center of Neurology and Psychiatry biobank. No financial incentives were provided to participants.
Ethics oversight	This study was approved by the Ethics Committee of Keio University School of Medicine (approval number: 20231158), and conducted in accordance with the Declaration of Helsinki and relevant guidelines.

Note that full information on the approval of the study protocol must also be provided in the manuscript.

Field-specific reporting

Please select the one below that is the best fit for your research. If you are not sure, read the appropriate sections before making your selection.

☒ Life sciences ☐ Behavioural & social sciences ☐ Ecological, evolutionary & environmental sciences

For a reference copy of the document with all sections, see nature.com/documents/nr-reporting-summary-flat.pdf

Life sciences study design

All studies must disclose on these points even when the disclosure is negative.

Sample size	Sample sizes were determined based on prior experimental experience, expected effect sizes, and feasibility constraints. Detailed sample numbers for each experimental group are provided in the figure legends and Methods.
Data exclusions	Pre-established criteria for exclusion were applied. In the rat miRNA-seq experiments, samples with miRNA concentrations below 20 pg/µL were excluded. In the human CSF dataset, two statistical outliers were excluded based on robust PCA and Mahalanobis distance thresholds. These criteria and excluded samples are detailed in Supplementary Table S2 and S4.
Replication	Key experiments, such as miRNA sequencing, were conducted once per group due to sample limitations, but included multiple biological samples per condition. Validation using qPCR was performed in biological replicates (n = 4 for rats). In vitro experiments were not replicated. Replicate numbers are detailed in the figure legends.
Randomization	In the animal experiments, rats were randomly assigned to either the spinal cord injury (SCI) group or the sham-operated group prior to surgery. Human clinical samples were assigned to groups (recovery, non-recovery, control) retrospectively based on predefined neurological outcomes (Frankel grade). In vitro experiments did not involve group randomization.

Blinding	Sample processing and data acquisition (e.g., EV isolation, RNA extraction, qPCR, sequencing) were performed in a blinded manner for both animal and human samples. Investigators were not blinded during data analysis due to study design constraints.

Reporting for specific materials, systems and methods

We require information from authors about some types of materials, experimental systems and methods used in many studies. Here, indicate whether each material, system or method listed is relevant to your study. If you are not sure if a list item applies to your research, read the appropriate section before selecting a response.

Materials & experimental systems

n/a	Involved in the study
☐	☒ Antibodies
☐	☒ Eukaryotic cell lines
☒	☐ Palaeontology and archaeology
☐	☒ Animals and other organisms
☐	☒ Clinical data
☒	☐ Dual use research of concern
☒	☐ Plants

Methods

n/a	Involved in the study
☒	☐ ChIP-seq
☒	☐ Flow cytometry
☒	☐ MRI-based neuroimaging

Antibodies

Antibodies used	The following primary antibodies were used for immunohistochemistry: anti-GFAP (rabbit polyclonal, Cat# 16825-1-AP, Proteintech; 1:1000), anti-HuC/D (mouse monoclonal, Cat# A-21271, Thermo Fisher Scientific; 1:500), anti-Olig2 (rabbit polyclonal, Cat# AF2418, R&D Systems; 1:200), and anti-Iba1 (rabbit polyclonal, Cat# 019-19741, FUJIFILM Wako; 1:500). Secondary antibodies included Alexa Fluor 488-conjugated goat anti-rabbit IgG (Cat# A-11008, Thermo Fisher Scientific; 1:1000) and Alexa Fluor 594-conjugated goat anti-mouse IgG (Cat# A-11005, Thermo Fisher Scientific; 1:1000). All antibodies used are commercially validated and showed expected staining patterns consistent with cell-type–specific localization.
Validation	All antibodies used in this study are commercially available and have been validated by the manufacturers for immunohistochemistry in rodent brain and spinal cord tissues. In our experiments, each antibody exhibited staining patterns consistent with known cell-type–specific localization (astrocytes, neurons, oligodendrocytes, and microglia), further supporting their specificity.

Eukaryotic cell lines

Policy information about cell lines and Sex and Gender in Research

Cell line source(s)	hiPSC line 201B7 (RRID: CVCL_A324) obtained from the Center for iPS Cell Research and Application (CiRA), Kyoto University, Japan. Day-14 motor-neuron derivatives were used.
Authentication	The identity of the human motor neurons was confirmed based on morphology and expression of motor neuron–specific markers, including HB9 and ChAT. No additional STR profiling was performed.
Mycoplasma contamination	The cultures were confirmed to be free of mycoplasma contamination by routine PCR testing.
Commonly misidentified lines (See ICLAC register)	The hiPSC line 201B7 (RRID: CVCL_A324) is not listed among commonly misidentified or cross-contaminated cell lines in the ICLAC database.

Animals and other research organisms

Policy information about studies involving animals; ARRIVE guidelines recommended for reporting animal research, and Sex and Gender in Research

Laboratory animals	Eight-week-old female Sprague-Dawley rats (150–200 g; Sankyo Labo Service Corporation, Inc., Tokyo, Japan) were used. Animals were housed under specific-pathogen-free conditions with ad libitum access to food and water.
Wild animals	Not applicable – no wild animals were used in this study.
Reporting on sex	Only female rats were used to reduce variability and avoid the confounding effects of hormonal cycling in spinal cord injury models.
Field-collected samples	Not applicable – no field-collected samples were used.
Ethics oversight	All animal experiments were approved by the Animal Care and Use Committee of Keio University (approval no. 13020) and conducted in accordance with institutional guidelines.

Note that full information on the approval of the study protocol must also be provided in the manuscript.

Clinical data

Policy information about clinical studies

All manuscripts should comply with the ICMJE guidelines for publication of clinical research and a completed CONSORT checklist must be included with all submissions.

Clinical trial registration	CSF samples were obtained from participants in a registered clinical trial (ClinicalTrials.gov identifier: NCT02193334).
Study protocol	The study protocol was approved by the Ethics Committee of Keio University (approval no. 20231158) and conducted in accordance with the Declaration of Helsinki. Participants provided written informed consent.
Data collection	CSF samples were collected from patients with acute spinal cord injury 72 hours post-injury as part of a previously published phase I/II clinical trial (Nagoshi et al., J Neurotrauma, 2020). Control CSF samples were provided by the National Center of Neurology and Psychiatry.
Outcomes	Patients were stratified into recovery and non-recovery groups based on neurological improvement from Frankel grade A at 72 hours to grades B or C at day 168 post-injury.

Plants

Seed stocks	Not applicable
Novel plant genotypes	Not applicable
Authentication	Not applicable
